# The *Nocardia cyriacigeorgica* GUH-2 genome shows ongoing adaptation of an environmental Actinobacteria to a pathogen’s lifestyle

**DOI:** 10.1186/1471-2164-14-286

**Published:** 2013-04-27

**Authors:** Anthony Zoropogui, Petar Pujic, Philippe Normand, Valérie Barbe, Patrick Belli, Arnault Graindorge, David Roche, David Vallenet, Sophie Mangenot, Patrick Boiron, Véronica Rodriguez-Nava, Sebastien Ribun, Yves Richard, Benoit Cournoyer, Didier Blaha

**Affiliations:** 1 Research group on “Bacterial Opportunistic Pathogens and Environment”, Université de Lyon, Lyon, France; 2 Research group on “Actinorhizal symbiosis”, Université de Lyon, Lyon, France; 3 Research group on “Environmental Microbiology Lyon – Biological Resource Center”, UMR5557 Ecologie Microbienne, Université de Lyon, Université Lyon 1, CNRS, and VetAgro Sup Veterinary Campus, Lyon, France; 4 Commissariat à l’Energie Atomique, Genoscope 91057, Evry cedex, France

**Keywords:** *Nocardia cyriacigeorgica*, Regions of genomic plasticity, Insertion sequences, COG, Evolution, Opportunistic pathogen

## Abstract

**Background:**

*Nocardia cyriacigeorgica* is recognized as one of the most prevalent etiological agents of human nocardiosis. Human exposure to these Actinobacteria stems from direct contact with contaminated environmental matrices. The full genome sequence of *N. cyriacigeorgica* strain GUH-2 was studied to infer major trends in its evolution, including the acquisition of novel genetic elements that could explain its ability to thrive in multiple habitats.

**Results:**

*N. cyriacigeorgica* strain GUH-2 genome size is 6.19 Mb-long, 82.7% of its CDS have homologs in at least another actinobacterial genome, and 74.5% of these are found in *N. farcinica*. Among *N. cyriacigeorgica* specific CDS, some are likely implicated in niche specialization such as those involved in denitrification and RuBisCO production, and are found in regions of genomic plasticity (RGP). Overall, 22 RGP were identified in this genome, representing 11.4% of its content. Some of these RGP encode a recombinase and IS elements which are indicative of genomic instability. CDS playing part in virulence were identified in this genome such as those involved in mammalian cell entry or encoding a superoxide dismutase. CDS encoding non ribosomal peptide synthetases (NRPS) and polyketide synthases (PKS) were identified, with some being likely involved in the synthesis of siderophores and toxins. COG analyses showed this genome to have an organization similar to environmental Actinobacteria.

**Conclusion:**

*N. cyriacigeorgica* GUH-2 genome shows features suggesting a diversification from an ancestral saprophytic state. GUH-2 ability at acquiring foreign DNA was found significant and to have led to functional changes likely beneficial for its environmental cycle and opportunistic colonization of a human host.

## Background

*Nocardia* is part of the well-known CMN actinobacterial group that also includes *Corynebacterium* and *Mycobacterium* in the *Corynebacteriales* order [1]. These Actinobacteria are characterized by long-chain mycolic acids in their cell wall [2], making them acid-resistant according to the Ziehl-Neelsen staining procedure, and favoring resistance to hydrophilic chemicals and dehydration. All CMN genera include pathogenic strains causing human diseases that affect millions of individuals such as leprosy, tuberculosis, and diphtheria. Besides, the CMN group also includes saprophytes that thrive in soils, waters, and polluted environments.

The *Nocardia* genus comprises about 80 species [3]. *N. cyriacigeorgica* was defined as a species in 2001 following the isolation and characterization of strain IMMIB D-1627 T from a bronchial discharge in a chronic bronchitis patient in Gelsenkirchen, Germany [4]. *N. cyriacigeorgica* can be differentiated from other species by 16S rDNA sequence analysis, their ability at growing on acetamide but inability at using proline as carbon and nitrogen sources. Definition of this species was confirmed by Conville *et al.* (2007) during their investigation of *Nocardia* strains with a type VI drug resistance pattern (characterized by a resistance to penicillins and a susceptibility to the broad-spectrum cephalosporins) [5]. *N. cyriacigeorgica* differs from *N. farcinica* strains by their ability at synthesizing a nitrate reductase and hydrolyzing xantine but their inability at synthesizing a urease, at hydrolyzing esculin and growing on L-rhamnose [6]. *N. cyriacigeorgica* strains were described as etiological agents of human pneumonia, brain abscesses, and kidney, heart and eye infections [4,7-12]. It is the most prevalent species involved in human nocardiosis in North America [11,13] and its prevalence in France was estimated at 12% among human nocardial infections declared between 2000 and 2007 [14]. Nocardiosis can be fatal for immunosuppressed individuals [13,14]. There is no report of *Nocardia* cross-contaminations in human populations, suggesting that environmental exposure is the main cause of infection. However, *N. cyriacigeorgica* has rarely been reported among environmental samples. Nevertheless, *N. cyriacigeorgica* strains have been recovered from oil contaminated soils [15,16], and were shown to oxidize a variety of aliphatic compounds [17].

In this work, the *N. cyriacigeorgica* GUH-2 genome sequence is presented, and compared with those of other Actinobacteria. The GUH-2 strain was isolated from a primary human kidney infection with systemic progression, which had a fatal outcome at Georgetown University Hospital, Washington, D.C [10]. The ability of this strain to induce Parkinson-like symptoms in inoculated mouse and monkey models [18,19] made it the model strain to study *Nocardia* biology and pathogenesis. These latter observations led to investigations on its possible role in some human Parkinson cases [20,21]. Animals infected by *N. cyriacigeorgica* were found to develop abnormal behaviors like rhythmic vertical “yes-yes” head–shaking movements, stooped posture, hesitation to move forward, retropulsion, and restlessness [22]. These parkinsonian-like symptoms appeared to be related to (i) a decrease in dopamine receptors and (ii) a programmed cell death of dopaminergic neurons within the *substantia nigra* in mice [23]. Intraperitoneal injection of antiparkinsonian drugs such as L-DOPA temporarily alleviated these symptoms [18,24]. *N. cyriacigeorgica* GUH-2 was also reported, in several independent experiments, to produce a substance(s) that can induce apoptosis and dopamine depletion [25,26]. Inferences on the likely nature of this substance(s) (probably a proteasome inhibitor) were made from the CDS sequence presented in this paper. A transposon mutagenesis screening of *Mycobacterium tuberculosis* showed the structural proteasomal genes of this closely related bacterium to be involved in their response toward oxidative and nitrosative stresses [27]. The nocardial proteasome could thus play a role in virulence.

The GUH-2 genomic sequence was also used to identify key evolutionary events in the emergence of *N. cyriacigeorgica*. Other members of the CMN group were shown to have evolved through important DNA reshuffling. Insertion sequences (IS) have largely contributed to genome rearrangements in the *Corynebacterium* and *Mycobacterium* genera, favoring deletion of genes, inversions, and functional specialization [28]. Important levels of CDS duplications and domains reshuffling were reported in Mycobacteria (50% of *M. tuberculosis* H37Rv) [29]. Statistical tests were performed to identify biases in *N. cyriacigeorgica* GUH-2 CDS and COG contents. A phylogenomic approach was developed to track the origin of some CDS or CDS clusters. These comparisons highlighted regions of genomic plasticity (RGP) among the *N. cyriacigeorgica* GUH-2 genome. Selection of these RGP was probably a driving force in the emergence of *N. cyriacigeorgica* GUH-2. These results revealed highly dynamic genomic evolutionary patterns in *N. cyriacigeorgica* caused by a significant ability at acquiring foreign DNA.

## Results

### Virulence status of *N. cyriacigeorgica* GUH-2

**Figure 1:**
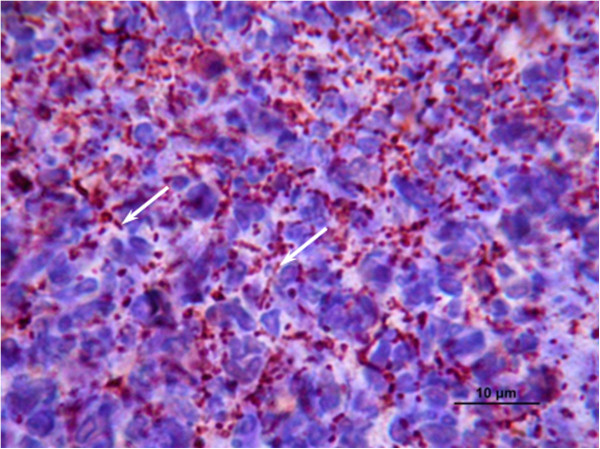
**Histological observations of mice tissue infected by*N. cyriacigeorgica*GUH-2.** Photograph illustrating the immunohistochemistry analysis of kidney cells from a case of fatal septicemia; white arrows indicate filamentous bacteria.

Virulence of *N. cyriacigeorgica* GUH-2 strain was confirmed by intravenous injection of approximately 10^7^ CFU in the tail of BALB/c mice. The death rate rose to 60% 7 days after infection. Autopsy indicated death to be due to septicemia with formation of nodules in several organs: kidneys, liver, brain, spleen, lungs and heart (Figure 1). Microscopic analysis of nodules showed high numbers of poly- and mono-nucleated inflammatory cells, and *N. cyriacigeorgica* GUH-2 cells. Some mice were injected a lower number (3.5 × 10^5^ CFU) of *N. cyriacigeorgica* GUH-2 cells and developed abnormal behavior: rigidity, stooped posture, hemiparesis and vertical yes-yes head shaking probably due to a brain infection.

### General features of the *Nocardia cyriacigeorgica* genome

The 6.19-Mb genome of *N. cyriacigeorgica* was sequenced and annotated during this work. It was found to harbor a single circular chromosome, and to have a ~68.4% G + C content. Three *rrn* operons containing the genes for the 16S, 23S and 5S rRNAs, 5,477 predicted protein-coding sequences (CDS), 49 tRNA genes, and 14 pseudogenes i.e. truncated genes, were detected. A function could be predicted for most CDS (62.23%). Other CDS were detected among other bacterial groups (28.10%) or showed no homology with known sequences (9.67%) (Table 1). *N. cyriacigeorgica* GUH-2 strain did not harbor a plasmid. The genome coding density was estimated at 86.73%, which is markedly lower than the 91% value observed in related genomes (Table 1).

**Table 1:** **Comparison of genomic features between *N. cyriacigeorgica *GUH-2 and eight Actinobacteria**

	***N. cyriacigeorgica***	***N. farcinica***	***R. equi***	***R. jostii***	***M. tuberculosis***	***M. smegmatis***	***C. diphtheriae***	***C. glutamicum***	***A. mediterranei***
**Features**	**GUH-2**	**10152**	**ATCC 33707**	**RHA1**	**H37Rv**	**MC2 155**	**NCTC 13129**	**ATCC 13032**	**U32**
Size (nt)	6,194,645	6,021,225	5,235,298	9,702,737	4,411,532	6,988,209	2,488,635	3,309,401	10,236,715
G + C (%)	68.37	70.83	68.81	67.0	65.61	67.40	53.48	53.81	71.29
Average CDS length (nt)	983.29	922.77	952.39	872.17	923.13	905.65	927.24	923.69	936.1
Average intergenic region (nt)	171.09	117.23	109.07	117.54	114.69	84.84	119.08	156.45	126.77
Protein coding density (%)	86.73	90.93	91.16	90.29	91.33	91.16	87.11	86.49	90.69
Protein-coding sequences (CDS)	5.491	5.984	5124	9.145	4.454	7.449	2.491	3.128	9.988
Pseudogenes	14	Un*	13	40	8	290	131	2 4	
rRNA (operon)	3	3	1	4	3	6	5	6	4
tRNA	49	53	52	52	45	47	54	60	52

**Figure 2:**
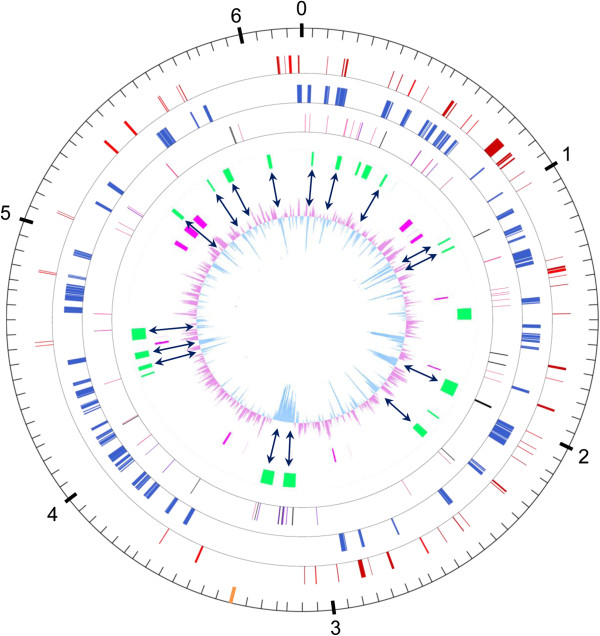
**Circular representation of the *N. cyriacigeorgica *chromosome.** Scale is in megabases and indicated on the outer black circle. The orange bar indicates position of the replication terminus. Black arrows show correspondence between RGP and low G + C content. Moving inward, the second circle indicates putative virulence genes (red); the third circle indicates conserved synteny groups (≥ 5 CDS) between *N. cyriacigeorgica*, *N. farcinica*, *R. jostii* and *M. tuberculosis* (blue); the fourth circle indicates tRNA genes (black), phage related genes (soft pink) and IS (purple); the fifth circle indicates selected regions of genomic plasticity i. e. RGP-Cy1 to RGP-Cy22 (green; also see Table 2); the sixth circle indicates the largest CDS observed (pink) and the seventh circle shows GC plot of the *N. cyriacigeorgica* genome.

GUH-2 chromosome harbors eleven insertion sequences (see IS section), and 15 CDS encoding putative phage proteins. Putative virulence genes are scattered along the chromosome without distinguishable pathogenicity island (see Additional file 1 for a complete listing). Several syntons of 5 CDS or more were found conserved between *N. cyriacigeorgica*, *N. farcinica*, *Rhodococcus jostii* and *Mycobacterium tuberculosis* (Figure 2). Non-conserved regions frequently showed distinct G + C % biases. Regions encoding the largest putative proteins from the genome were found to have features of non ribosomal peptide synthetases (NRPS) and showed a high G + C content. The only exception is *NOCYR_4710* CDS which has a G + C content slightly lower than the averaged one, and encodes a putative Dipeptidyl carboxypeptidase Dcp located near a transposase-related CDS (Figure 2).

**Figure 3:**
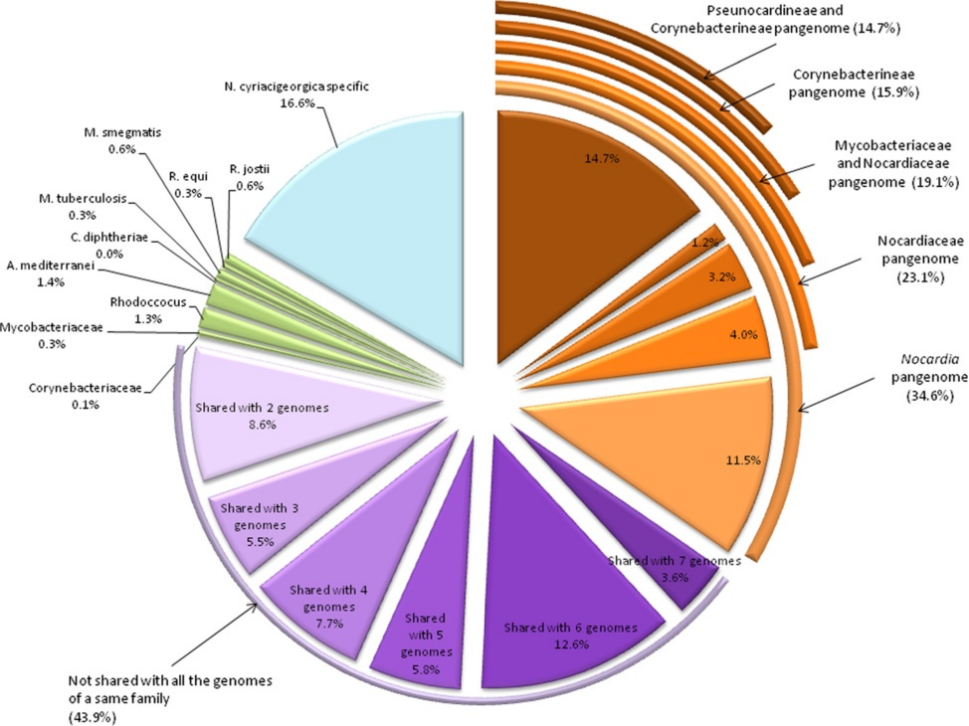
**Percentage of *N. cyriacigeorgica *CDS shared with eight selected Actinobacteria genomes (*A. mediterranei*, *C. diphtheriae*, *C. glutamicum*, *M. tuberculosis*, *M. smegmatis*, *N. farcinica*, *N. cyriacigeorgica*, *R. equi*, and *R. jostii*).** CDS belonging to pangenomes are in orange and were related to phylogenetic suborders and families shown in Figure 4. *N. cyriacigeorgica* CDS shared by two to seven Actinobacteria belonging to different families (Nocardiaceae, Mycobacteriaceae, Corynebacteriaceae, Pseudonocardiaceae) are in purple, CDS shared with only one genome are in green and *N. cyriacigeorgica* specific CDS are in blue (threshold of 40% identity).

**Figure 4:**
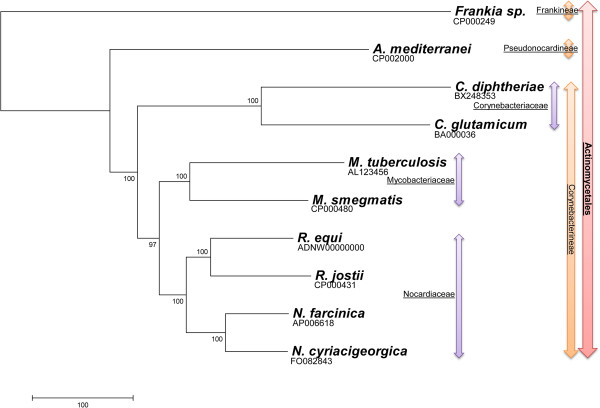
**NJ phylogenetic tree of the Actinobacteria inferred from concatenated *gyrB-rrs-secA1-hsp65-rpoB *DNA sequences.** Phylogenetic order, suborders and families are indicated in red, orange and purple respectively.

To better understand the events that had led to the present-day *N. cyriacigeorgica* GUH-2 genomic structure, its core genome was delimited by identifying CDS conserved in a panel of closely related Actinobacteria (Figure 3). 15% (805 of 5477) of the CDS was found in a putative common ancestor to all genomes except *Frankia sp.* CcI3 (Figure 4). Of these CDS, 80% could be assigned a function and, as expected, a large proportion was inferred to play part in basic bacterial functions such as synthesis of proteins, nucleosides and nucleotides, amino acids, co-factor prosthetic groups and carriers, and of the cell envelope (see Additional file 2 for the full listing). Interestingly, the only CDS exclusively shared between *N. cyriacigeorgica* and *M. tuberculosis* were those of IS987, an insertion sequence, suggesting a likely transfer of this IS between these species. The *N. cyriacigeorgica* and *N. farcinica* genomes were found to share 74.5% of their CDS, delimiting a *Nocardia* pangenome of about 4.5 Mb. 1398 CDS (25.5%) of *N. cyriacigeorgica* were not found in the *N. farcinica* genome. Most of these CDS are of unknown function (78%) but some are likely involved in phosphonate transport and metabolism (phytase), synthesis of fatty acids, glutamate metabolism, nitrite/nitrate transport, and RuBisCO production. Conversely, 2253 CDS of *N. farcinica* were not found in the *N. cyriacigeorgica* genome (data not shown). Again, a high proportion of these CDS (69%) were “unk” CDS (unknown function) and a few could be related to particular activities like synthesis of thiocyanate (toxic compound) (4 CDS), catabolism of urea (6 CDS), of auxins (6 CDS) and lignin, heavy metal resistance and virulence (9 CDS). These differences were in line with biochemical tests such as measurement of nitrate reductase and urease activities, performed to differentiate these two species. 11.6% CDS (633 out of 5477) of *N. farcinica* and *N. cyriacigeorgica* genomes were not recorded in other actinobacterial sequenced genomes (Figure 3). These two *Nocardia* strains have an equivalent genome size which is longer than the ones of primary actinobacterial pathogens (Table 1). 16.6% (777 kb) of *N. cyriacigeorgica* CDS content was not recorded in any other organisms referenced in the databases. These CDS were sometimes related to mobile and extrachromosomal elements but most of them were of “unknown function”. A search for amplified CDS among *N. cyriacigeorgica* genome revealed 161 occurrences, of which 132 are duplicated, 21 are triplicated and 8 are quadruplicated. More than 29% of these CDS could not be given a particular function, and 79% were also found in *N. farcinica*. IS (1 CDS), *mce* (4 CDS), transcriptional regulators (12 CDS) and nitrate reductase (6 CDS) were part of these amplified regions. More than 23% of these CDS were found among regions of genomic plasticity (RGP) (see Additional file 3 for more details).

**Figure 5:**
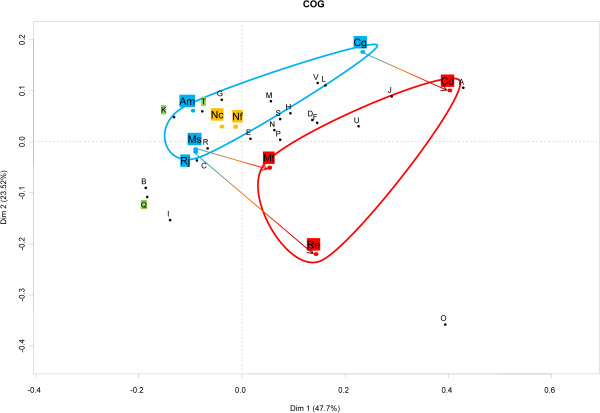
**Correspondance analysis of COGs in the genomes of *Nocardia cyriacygeorgica *and relatives identified on the Mage platform.** COGs were retrieved for (Am) *A. mediterranei*, (Cd) *C. diphtheriae*, (Cg) *C. glutamicum*, (Mt) *M. tuberculosis*, (Ms) *M. smegmatis*, (Nc) *N. cyriacigeorgica*, (Nf) *N. farcinica*, (Re), *R. equi,* (Rj) *R. jostii*. The pathogens are highlighted in red, the non-pathogens or saprophytic bacteria in blue and the *Nocardia* in orange. The COGs comprise (**A**) RNA processing and modification, (**B**) Chromatin structure and dynamics, (**C**) Energy production and conversion, (**D**) Cell cycle control, cell division, chromosome partitioning, (**E**) Amino acid transport and metabolism, (**F**) Nucleotide transport and metabolism, (**G**) Carbohydrate transport and metabolism, (**H**) Coenzyme transport and metabolism, (**I**) Lipid transport and metabolism, (**J**) Translation, ribosomal structure and biogenesis, (**K**) Transcription, (**L**) Replication, recombination and repair, (**M**) Cell wall/membrane/envelope biogenesis, (**N**) Cell motility, (**O**) Posttranslational modification, protein turnover, chaperones, (**P**) Inorganic ion transport and metabolism, (**Q**) Secondary metabolites biosynthesis, transport and catabolism, (**R**) General function prediction only, (**S**) Function unknown, (**T**) Signal transduction mechanisms, (**U**) Intracellular trafficking, secretion, and vesicular transport, (**V**) Defense mechanisms. The first two principal components that represent respectively 47.7% (horizontal axis) and 23.5% (vertical axis) of the total variance of the dataset are plotted against one another.

Out of the 5477 CDS present in *N. cyriacigeorgica* genome, 4016 CDS (i. e. 73%) could be assigned to a COG [see Additional file 4]. The proportion of these COGs among Actinobacteria was similar, with a slightly higher occurrence of CDS among the “transcription” (K) and “signal transduction” (T) COGs of the *Nocardia* genomes. Correspondence analysis of the number of CDS per COG among a set of actinobacterial species was performed, to identify a possible bias related to the pathogenic nature of the species. This analysis showed that the number of CDS per COG could differentiate primary pathogens from non-pathogens. This “pathogen pattern” was more significant than the COG organization bias inferred from species belonging to a same genus. On the other hand, all non-pathogens had closer COG patterns even though some were part of different genus or part of a genus showing pathogenic species. COG patterns of the *Nocardia* genomes were found similar to those of non-pathogens (Figure 5, but also see the Additional file 4). A correspondence analysis on the functional domains inferred from the CDS of the “transcription” COG was performed (Additional file 5 and Additional file 6). This analysis did not segregate the dataset according to the pathogenic nature of the species regardless of their genus. Instead, the numbers of CDS per sub-division were found similar between the *Nocardia* genomes and similar to those observed among *M. tuberculosis.*

### Regions of genomic plasticity

**Figure 6:**
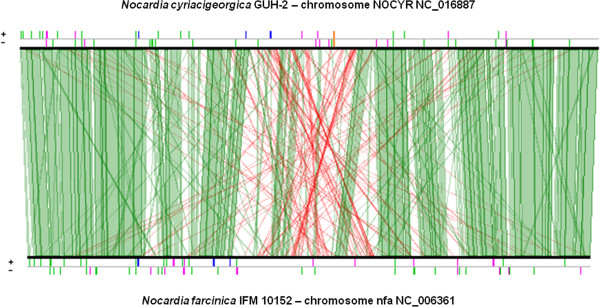
**Lineplot based on conserved synteny results (≥ 5 CDS) between *N. cyriacigeorgica *and *N. farcinica *genomes.** Strand conservations (in green) and strand inversions (in red) are shown. Above the lineplot, orange bar indicates approximate terminus replication location and pink bars indicate transposases and insertion sequences. Blue bars indicate rRNA and green ones tRNA.

A lineplot graph between *N. farcinica* and *N. cyriacigeorgica* genomes, representing synteny results of series of 5 CDS or more, was performed in order to visualize the distribution of variable and conserved regions (Figure 6). This analysis showed the number of conserved CDS between these chromosomes to increase towards their respective origin of replication. The lowest concentration of these CDS was observed around the chromosomal terminus of replication. Overall, the organization of these variations was quite similar between the two halves of the circular chromosome creating a mirror-like effect indicative of increasing evolutionary constraints from the terminus towards the origin of replication (on both strand). The chromosomal terminus is partially visible on the circular map of *N. cyriacigeorgica* genome, and shows a large segment with a distinct G + C content according to the GC plot (Figure 2). The Artemis Comparison Tool (ACT) was used to refine these analyses and identify DNA segments >4.5 kb or containing more than 5 CDS. These DNA regions were not showing all the features of genomic islands such as a tRNA gene at one end, an integrase CDS or a G + C bias distinct from the one of the genome. Twenty-two RGP could be detected using this approach (named RGP-Cy#) (Table 2). Twenty-one of these RGP were also detected with the *RGPfinder* tool of the MaGeplateform. RGP-Cy6 was not detected in this latter analysis because of its length of 4.9 kb. Of these RGP, four could be considered genomic islets (< 10 kb), and the largest RGP was of about 80 kb. These RGP represented a total of 704 kb i.e. 11.4% of the genome and encoded 622 CDS. The average G + C% content of these RGP is of 65.5%, with values ranging from 60.1% to 68.6%. RGP boundaries were analyzed. tRNA or tmRNA genes were detected at the extremity of ten of these. Direct DNA repeats were observed for RGP-Cy8 from positions 1098658 to 1098672 and 1109676 to 1109690, at its left and right ends, respectively. Four RGP showed IS sequences. Five RGP contain CDS implicated in DNA modification processes such as integrases, recombinases, endonucleases and excisionases. These CDS were probably involved in the acquisition of these RGP. Ten RGP did not show any of the above features. Most CDS on these selected RGP encode putative proteins and transcriptional regulators of unknown function (74%). Some CDS, likely representing a benefit for *N. cyriacigeorgica* GUH-2, could be identified and predicted to encode a catalase (RGP-Cy3), a limonene monooxygenase (RGP-Cy16), and a sulfonate ABC transporter (RGP-Cy21). Furthermore, CDS involved in nitrate metabolism were identified on the RGP-Cy14. A cobalamin-independent methionine synthase (*metE*) and three CDS involved in citrate metabolism were found on RGP-Cy15 while *pglY* and *pglZ* involved in phage defense were found on Cy10 (Table 2).

**Table 2:** **Regions of genomic plasticity (RGP-Cy#) identified in *N. cyriacigeorgica *by ACT comparisons with the *N. farcinica *genome**

**RGP**	**GI position**	**Virulence gene (in or around GI)**	**ORF begin**	**ORF end**	**Length (kb)**	**Nb. of genes**	**Gene class**	**Putative role of GI**	**tRNA**
Cy1	61471..73200		*nocyr_0048*	*nocyr_0063*	11.7	14	Unknown	Unknown	0
Cy2	213775..239600		*nocyr_0182*	*nocyr_0205*	25.8	21	Enzymes, transporters, histidine degradation, CSP	Unknown	3
Cy3	343550..357558	*nocyr_0300*	*nocyr_0300*	*nocyr_0309*	14	10	Enzymes, regulators, transporters, catalase	Adaptation to atypical conditions	1
Cy4	387100..424100		*nocyr_0340*	*nocyr_0383*	37	43	Recombinase, integrase, topisomerase, ADN pol III, cadmium inductible protein	Plasticity, adaptation	1
Cy5	512800..526200		*nocyr_0472*	*nocyr_0488*	13.4	17	regulators, transporters	Unknown	0
Cy6	594300..599228	0547 → 0555, 0566 → 0567	*nocyr_0557*	*nocyr_0561*	4.9	5	Regulators, **ISNcy5**	Plasticity	1
Cy7	1045000..1055000		*nocyr_0920*	*nocyr_0925*	10	6	Unknown	Unknown	1
Cy8	1098658..1109690		*nocyr_0967*	*nocyr_0978*	11.3	11	Integrase, endoribonuclease	Plasticity	0
Cy9	1480355..1548800		*nocyr_1341*	*nocyr_1391*	69.3	51	Enzymes, regulators, mycosin	Virulence	2
Cy10	1937100..2016932	*nocyr_1792*	*nocyr_1739*	*nocyr_1800*	79.8	68	Enzymes, regulators, C31 phage resistance genes	Defense	1
Cy11	2164650..2173100		*nocyr_1932*	*nocyr_1940*	8.4	9	Unknown	Unknown	0
Cy12	2273150..2315500		*nocyr_2044*	*nocyr_2082*	42.4	36	Enzymes, transporters, regulators, integrases	Plasticity	1
Cy13	3131000..3204200		*nocyr_2827*	*nocyr_2884*	73.2	58	Enzymes, regulators, **ISNcy2-b**, **ISNcy4**, integrases, PBP	Plasticity, adaptation	0
Cy14	3265400..3337600		*nocyr_2933*	*nocyr_2985*	72.2	61	Enzymes, Nitrate reduction/expulsion, **ISNcy1-d**, regulators, recombinase	Nitrite/Nitrate metabolism, plasticity	1
Cy15	4299671..4308500		*nocyr_3906*	*nocyr_3911*	8.8	6	Cobalamin and citrate metabolism	Vit B12, energy metabolism	0
Cy16	4339344..4363500		*nocyr_3940*	*nocyr_3962*	24.2	23	Enzymes, regulators, limonene-momooxygenase	Unknown, Adaptation	0
Cy17	4409577..4447497		*nocyr_4002*	*nocyr_4035*	37.9	34	Enzymes, regulators, transporters	Unknown	0
Cy18	4523400..4592400	*nocyr_4112, nocyr_4128*	*nocyr_4097*	*nocyr_4162*	69	66	Enzymes, regulators, NRPS, transporters, fatty acid synthesis, spermidin, sigma factor, DHB	Fatty acid and peptide synthesis, adaptation, virulence, plasticity	1
Cy19	5338862..5361100		*nocyr_4801*	*nocyr_4818*	22.2	14	Enzymes, regulators	Unknown, vitamine métabolism	0
Cy20	5611900..5622135		*nocyr_5027*	*nocyr_5036*	10.2	10	Unknown	Unknown	0
Cy21	5720300..5758400	*nocyr_5135, nocyr_5136*	*nocyr_5133*	*nocyr_5170*	38.1	38	Enzymes, transporters, regulators, sulfur metabolism	Energy metabolism, virulence, adaptation	0
Cy22	5992000..6012890		*nocyr_5383*	*nocyr_5404*	20.9	21	Enzymes	Unknown	0

**Figure 7:**
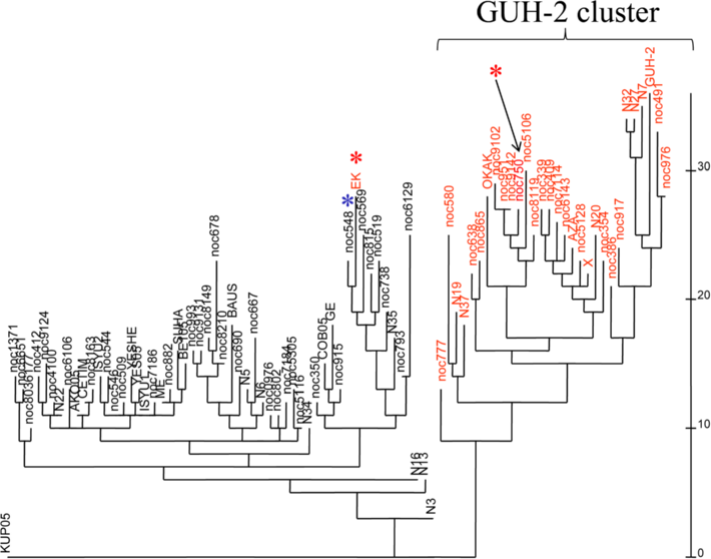
**Cladogram illustrating the distribution of 22 RGP observed in the *N. cyriacigeorgica *GUH-2 genome among a panel of 83 *N. cyriacigeorgica *strains.** PCR screenings targeted three markers among each of the 22 RGP. Strains indicated in red harbored 5 or more RGP and those in black harbored less than 5 RGP. “*” indicates strains moving from one cluster to another depending on the number of markers analyzed per RGP. The scale indicates the number of changes in the RGP patterns between pairs of strains.

PCR screenings were designed to investigate the distribution of these RGP among 83 *N. cyriacigeorgica* strains (Additional file 7). Prevalence of these RGP was quite variable, with some not being detected in other strains, and some being found among up to 69% of the strains. RGP-Cy4 and RGP-Cy8 were only found in the *N. cyriacigeorgica* GUH-2 genome and showed all the features of mobile elements. A cladogram was built using the RGP distribution patterns based on the positive and negative PCR results (Figure 7). Dataset of inner RGP and RGP-ends DNA targets were analyzed separately or together, and gave similar cladograms. All strains harboring 5 or more RGP were grouped in the “GUH-2 complex”. Other strains belonged to a “type strain complex”. In fact, RGP patterns of 30 strains were found to be in the “GUH-2 complex”, while patterns of 56 strains were allocated to the “type strain complex”. Among the “GUH-2 complex”, some of the selected RGP were highly prevalent: RGP-Cy1, RGP-Cy6, RGP-Cy11, RGP-Cy15, RGP-Cy16, RGP-Cy18 and RGP-Cy21 were found in 23, 18, 25, 29, 23, 20, and 26 strains respectively. Strain N7 harbored the highest number of RGP found in the *N. cyriacigeorgica* GUH-2 genome (15/22 positive PCR products). Among the type strain complex, RGP- Cy2, -Cy5, -Cy6, -Cy7, -Cy9, -Cy10, -Cy16, -Cy18, -Cy19, -Cy20, and -Cy21 were not recorded. No RGP seemed to be representative of this latter complex, and RGP-Cy3, RGP-Cy15 and RGP-Cy17 were the most prevalent. RGP-Cy3 was the most broadly distributed (69 positive strains) among the *N. cyriacigeorgica* species. PCR screenings targeting the left and right ends of RGP were defined to estimate the level of conservation of their respective proximal DNA region. The left (L) and right (R) ends of RGP-Cy9, -Cy15, and -Cy16 were broadly detected whereas only one end could be detected for RGP-Cy2 (R), -Cy3 (L), -Cy4 (R), -Cy7 (R), -Cy11 (R), -Cy17 (R), -Cy18 (L), -Cy19 (R), -Cy20 (L), -Cy21 (L), and -Cy22 (R) among all the strains tested. The L and R ends of the RGP-Cy5, Cy6, Cy10, and Cy11 were detected among the “GUH-2 complex”, while only RGP-Cy1 (L), -Cy3 (R), -Cy7 (L), -Cy11 (L), -Cy13 (L), -Cy20 (R), -Cy21 (R) could be detected among the “GUH-2 complex” (data not shown). It is noteworthy that an attempt was made at comparing this RGP classification with phylogenetic relationships inferred from 16S rDNA sequences. However, significant sub-groups among *N. cyriacigeorgica* could not be resolved with this marker (data not shown).

RGP represent gains in CDS likely to confer novel properties but CDS losses can also represent adaptations to particular habitats. 193 *N. farcinica* CDS were found mISing in the *N. cyriacigeorgica* GUH-2 genome. Among these, 35% encode putative proteins of unknown function but four clusters of CDS involved in glutamine metabolism, in phenyl acid acetic degradation, in thiocyanate degradation and nitrogen metabolism, and in urease synthesis were found mISing (see Additional file 3 for further details).

### The particular case of insertion sequences (IS)

On the *N. cyriacigeorgica* genome, eleven IS belonging to five different families were detected (IS*3*, IS*21*, IS*200*, IS*256* and IS*NCY*). A re-evaluation of the *N. farcinica* IS genome content was performed, and led to the identification of fifteen IS belonging to five families (IS*3*, IS*5*, IS*200*, IS*481*, and IS*630*) (Additional file 8). Only ISNfa14 and ISNcy8 of the IS*3* family were found located in the same DNA site of these two actinobacteria. These IS share 86% DNA identity. *N cyriacigeorgica* genome showed DNA signatures of two Tn3 transposons but one appeared to be truncated. *N. farcinica* genome also showed the presence of a Tn*3* in which the transposase CDS shares 85% DNA identity with the one of *N. cyriacigeorgica* transposon 2. Inverted repeats (IR) and/or direct repeats (DR) were identified for five *N. cyriacigeorgica* IS and for three *N. farcinica* IS. ISNcy2 copies (with DNA identities going from 82 to 100%) in *N. cyriacigeorgica* genome were found related to IS987 (75% DNA identity) which was only previously detected in the *M. tuberculosis* and *M. bovis* genomes. The *N. cyriacigeorgica* GUH-2 genome harbors the ISNcy1 in four copies with one being partial and three being identical. Three IS were found in several copies in *N. farcinica*. ISNfa1 of the IS*481* family was found in two copies with 99% DNA identity; ISNfa2 of the IS*5* family in eight copies with identities going from 72% to 99%; and ISNfap1, a partial element of the Tn*3* family, having 85% DNA identity with a copy found on *N. farcinica* larger plasmid.

The distribution of six of these IS/Tn elements was investigated among a sub-set (n = 18) of the *N. cyriacigeorgica* strains screened in the RGP section, a set (n = 11) of *N. farcinica* strains, and a panel of *Nocardia* species type strains (n = 12) (Additional file 9). Only ISNcy4 (Tn*3*) and ISNfa2 (IS*427* of the IS*5* family) were detected among both *N. cyriacigeorgica* and *N. farcinica* but their prevalence was quite different from one species to another. In fact, both of these elements were more prevalent among the panel of strains of *N. farcinica* than *N. cyriacigeorgica* strains selected for this study. ISNcy2 (IS*51* of the IS*3* family) was the most prevalent among *N. cyriacigeorgica* but was absent from the *N. farcinica* strains tested. This ISNcy2 was also found among the *N. otitidiscaviarum* type strain. ISNcy3 was not detected in *N. farcinica*. Its prevalence was higher among the GUH-2 complex of *N. cyriacigeorgica* (which was defined according to the distribution of RGP in the section above). A similar situation was observed for ISNcy4. ISNcy2 and ISNcy3 were not detected among the *Nocardia* type strains that were selected for this analysis, suggesting a distribution restricted to *N. cyriacigeorgica*. ISNfa1 and ISNfa5 were not detected in *N. cyriacigeorgica* and appeared to be restricted to *N. farcinica* strain 10152. ISNcy4 (Tn*3*) and ISNfa1 (IS*481*) share 89% identity, and were detected among several strains of *N. farcinica* and *N. cyriacigeorgica*. A cladogram was built from this IS distribution pattern analysis and was found to match the RGP distribution patterns (data not shown).

### Virulence-related functions

Several genes were previously found involved in virulence among Actinobacteria. Blast and keyword-based searches allowed identification of some of these CDS among the *N. cyriacigeorgica* genome. Six complete *mce* (mammalian cell entry) loci containing *yrbE* and *mas* (*mce* associated) CDS were found. Analysis of CDS encoding cell wall components involved in virulence revealed 85-kDa antigens family proteins (4 CDS), lipoproteins (19 CDS) and PE_PGRS/PPE family proteins (5 CDS). Two superoxide dismutase CDS (*sod*) and three catalase ones were also identified. Nitrate reductase CDS (*narBGHIJKY*, and *nirBD*), trehalose 6,6’-dimycolate transferase (one CDS) and RuBisCO (2 CDS) were detected and found to be clustered.

Extracellular enzymes were identified by searching for particular domains including a peptide leader and low number of transmembrane domains. The inferred secretome of *N. cyriacigeorgica* GUH-2 was compared with the ones of other Actinobacteria, and showed a majority (70%) of putative proteins of unknown function, several proteases, lipases as well as a transcriptional regulator and members of the *mce* genes (Additional file 10 shows these exported CDS in more detail). Comparison of the putative proteins of the *N. cyriacigeorgica* and *N. farcinica* secretomes showed high identities (between 81 to 86%), much higher than those observed with other Actinobacteria (60%).

The *N. cyriacigeorgica* GUH-2 genome shows a good potential for the synthesis of a number of metabolites that could be antimicrobials or proteasome inhibitors. Seven CDS were predicted to encode polyketides synthases (PKS), and 17 CDS were predicted to encode NRPS (non-ribosomal peptide synthetases). Among *N. farcinica*, 4 PKS and 15 NRPSs were found including the cluster of CDS previously described as producing a lipid-soluble iron-binding nocobactin. A cluster of nine CDSs similar to the coelibactine synthesis cluster in *Streptomyces coelicolor* was also identified in *N. cyriacigeorgica* GUH-2’s genome. *NOCYR_4800* was found to be the longest CDS of the genome (43689 pb) and likely to encode a NRPS with twelve modules. Two other NRPS operons containing two and three CDS could synthesize molecules containing thirteen and eleven building blocks, respectively. A 2-amino-9,10 epoxi-8-oxodecanoic acid was predicted for *NOCYR_0751*. This putative acid shows a structure similar to the epoxomicin proteasome inhibitor (epoxy group) but chemical assays will be required to confirm its synthesis. Other softwares did not find this structure.

**Figure 8:**
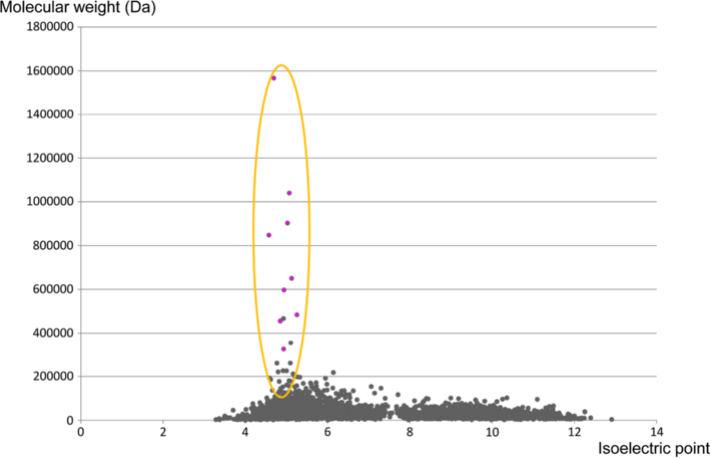
**Relation between isoelectric point (x-axis) and molecular weight (y-axis) of *N. cyriacigeorgica *proteins.**

It is noteworthy that a correlation was observed between the isoelectric point and the molecular weight of the inferred proteins of the *N. cyriacigeorgica* GUH-2 genome. The largest proteins of the genome, constituted mainly by NRPS and PKS, showed similar isoelectric points, suggesting a compartmentalization inside the cytosol that could allow a coordinated functioning of these enzymes (Figure 8).

### Genetic potentials and metabolic profiling

Phenotypic microarray datasets including antibiotic resistances were compared with the functional predictions made from the annotated *N. cyriacigeorgica* GUH-2 genome. Phenotypic profiling was performed by testing *N. cyriacigeorgica* ability to grow with various carbon and nitrogen sources. KEGG pathways were used to find the CDS involved in these pathways but also as a reference for explaining an absence of growth under certain conditions. (Additional file 11). Only seven amino acids (L-asparagine, L-aspartic acid, L-cysteine, L-glutamic acid, L-glutamine, L-histidine, L-lyxose, L-tyrosine) could be used as carbon and/or nitrogen sources. The absence of some CDS previously shown in other bacteria to be involved in the substrate catabolism or transport could explain several negative results. However, the lack of growth with L-aspartic acid, L-lysine and L-proline could not be explained by the absence of particular CDS.

The osmoadaptation capacity *N. cyriacigeorgica* GUH-2 was also tested and found to be high under high salt concentrations. This property could involve CDS encoding transport proteins and/or osmoprotectants like ectoine and betaine (Additional file 11). Growth tests were also performed under various pH. *N. cyriacigeorgica* GUH-2 was shown to grow at pH 7 and 8 but not pH 5.2. This dataset was completed by an analysis of the antibiotics resistance pattern of *N. cyriacigeorgica* GUH-2 using plate assays. *N. cyriacigeorgica* GUH-2 was confirmed to have a type VI pattern with resistance against several sulfonamides, aminoglycosides, tetracyclines and penicillins. It was found sensitive to cefamandole, cefotaxime, amikacin, and imipenem. Many CDS likely playing part in these antibiotic resistances were found in the *N. cyriacigeorgica* genome. A mutation in the gyrase A gene that can prevent ciprofloxacin binding, was detected. Several CDS were inferred as encoding β-lactamases in the GUH-2 genome, and could be responsible for the observed ampicillin, carbenicillin, oxacillin and penicillin resistances. The paromomycin and tobramycin resistances observed can be due to the expression of one of the three aminoglycoside phosphotransferases detected by the annotation process. Macrolide resistances could involve one of the six ribosomal RNA methyltransferases encoded by the GUH-2 genome. These may cause a decrease in the affinity of macrolides for the 50S ribosomal unit. The fourteen drug or multidrug efflux transporters encoded by this genome could be involved in tetracycline, penimepicycline, polymyxin B, paromomycin, D,L-serine hydroxamate, sisomicin, sulfamethazine, novobiocin and/or sulfadiazine resistance (data not shown) by extruding these molecules.

## Discussion

*Nocardia cyriacigeorgica* is an opportunistic pathogen causing many infections including deadly brain abscesses and granulomatous diseases among immune-compromised and healthy individuals. However, the bacterial properties involved in these infections are poorly understood. Here, the content, organization, plasticity, and functional potentialities of *N. cyriacigeorgica* GUH-2 full genome sequence are presented. A particular attention was paid to the analysis of CDS likely involved in virulence including antibiotic resistances. These analyses led to the identification of several RGP and IS elements that were then tracked among a panel of *N. cyriacigeorgica* strains. These screenings revealed an important ability of this species at acquiring DNA by horizontal transfer events.

### Virulence determinants

The *N. cyriacigeorgica* GUH-2 genome analyses revealed several genetic determinants related to virulence. Some of these are part of RGP but some were also part of the *Nocardia* or Actinobacteria pangenomes inferred from comparisons with the full genome sequence of *N. farcinica* and other Actinobacteria. The genome of *N. cyriacigeorgica* harbors virulence-related CDS such as Mce coding genes described as important virulence factors of *M. tuberculosis*[30]. Mce can act as transmembrane transporters favoring macrophage invasion [31]. These CDSs are organized in operons containing two *yrbE* CDS followed by six *mce* CDS and sometimes two *mce* associated (*mas)* CDSs. There are six copies of the complete *mce* operon in *N. cyriacigeorgica* and *N. farcinica* whereas four copies are identified in *M. tuberculosis* genome. Although, the importance of these CDSs in *M. tuberculosis* virulence has been shown [32], their detection in multicopies among *M. smegmatis* and *R. jostii* (six and four clusters, respectively) suggests a function not limited to cell entry [33]. Having access to the *N. cyriacigeorgica mce* CDS will allow transcriptomics experiments to identify the *mce* CDSs which are turned on during host cell colonization.

Other *N. cyriacigeorgica* CDSs besides *mce* have been described as important in the infection and cell invasion processes. In particular, the expression of superoxide dismutase and catalase CDS were observed during *N. cyriacigeorgica* macrophage invasion and were suggested to be involved in resistance towards oxidative stresses [34]. Two *sod* and three *kat* CDS were found in the *N. cyriacigeorgica* genome. The *katA* CDS is harbored by the RGP-Cy3 but the other two *kat* CDSs are found on conserved genomic regions. Other CDSs can also have a complementary action during *Nocardia* growth in macrophages. The trehalose 6,6’-dimycolate transferase CDS was found related to the 85-kDa antigen family protein [35] that can promote *M. tuberculosis* survival in macrophages by decreasing both phagosomal acidification and phagolysosomal fusion [36,37]. An encoded *N. cyriacigeorgica* hemolysin which can disrupt the phagolysosome membrane was also annotated and could also favor survival in macrophages [38,39]. The isocitrate lyase CDS observed on RGP-Cy15 could also be part of the *N. cyriacigeorgica* macrophage invasion process by preventing host cell apoptosis as observed in *M. tuberculosis*[40]. An intracellular pathogen not only requires defense mechanisms against macrophage antibacterial processes but also needs to survive under low oxygen pressure such as the one observed in poorly irrigated tissue of the mammal body [41]. CDS involved in such processes can thus also be defined as virulence-related determinants. *N. cyriacigeorgica* contains denitrification CDSs (*narBGHIJKY*, and *nirBD*) of which five are harbored by RGP-Cy14. *N. cyriacigeorgica* was initially described as an obligate aerobe but presence denitrifying CDS suggests an ability to grow under anaerobic conditions. However, the conditions allowing growth under low oxygen pressure remain to be defined.

Several metabolites, proteins, enzymes and lipids which are not directly involved in colonization but are at the frontline during host infection can also play a role in virulence such as cell wall constituents and some extracellular enzymes/metabolites. The cell wall is a protection for bacteria but can also be a target for the immune system. For example, PE/PPE serine α/β hydrolases membrane proteins are important in *Mycobacterium* pathogenic species to avoid detection or killing during their intracellular life in a variety of host cells [42,43]. However, their low number in *N. cyriacigeorgica* and *N. farcinica* could be an effect of their opportunistic status (environmental cycle) and could explain their poor ability to escape the immune system of a healthy host. *Nocardia* can also secrete several enzymes that may interfere with the host cell metabolism [25], in particular SODs [44], lipases [45] and proteases [46]. CDSs encoding such enzymes were observed in the *N. cyriacigeorgica* GUH-2 genome. Eight CDSs were found encoding extracellular lipases and 10 encoding extracellular proteases. Nevertheless, much more CDSs encoding putative secreted enzymes have been reported in *Mycobacterium* such as the ESAT (early secreted antigenic target) proteins [47]. *N. cyriacigeorgica* GUH-2 genome harbored three ESAT CDSs.

Another major group of secreted molecules that can play a role in virulence are siderophores. *Nocardia* strains can produce several siderophores like formobactin [48], amamistatin [49], brasilibactin [50], asterobactin [51] and nocobactin [52]. Nocobactin synthesis was previously found encoded by two genetic clusters, with cluster I positioned 195 kb from cluster II. The *N. cyriacigeorgica* GUH-2 genome showed a cluster I organization different from the one observed in the *N. farcinica* genome. The gene *nbtH* is absent in the *N. cyriacigeorgica* GUH-2 genome and replaced by a putative formyltransferase CDS, which could play a role in preventing the transfer of an acyl chain to the ϵ-amino group of lysine. The *N. cyriacigeorgica* GUH-2 cluster II shows high identities with the one of *N. farcinica* but additional CDS encoding NRPS and an exported protein of unknown function were recorded. The NRPS CDSs could play a role in the synthesis of this siderophore and change some of its properties. A coelibactin-like siderophore is also likely to be produced by *N. cyriacigeorgica* GUH-2. A conserved synteny was observed with *S. coelicolor* CDS involved in its synthesis except for a supplementary CDS encoding a cytochrome P450 protein.

These Actinobacteria are also known to synthesize other extracellular metabolites through NRPS and PKS-related processes. The *N. cyriacigeorgica* GUH-2 genome was found to encode 12 PKS-related CDSs and 22 NRPS-related ones. It is considered that the substance produced by *N. cyriacigeorgica* GUH-2 which can cause brain damages by inducing apoptosis and a dopamine depletion would be encoded by a NRPS or PKS [25,26]. Here, a putative metabolite produced by an operon of three NRPSs could be predicted to have a structure similar to the one of epoxomicin [53]. Such molecules have epoxy groups that could inhibit the functioning of proteasomes. NRPS and PKS are also involved in the synthesis of antibiotics, and could have been involved in the synthesis of transvalencin Z [54], DA-7218 [55], and nocardithiocin [56]. However, the molecules produced by these synthases are often difficult to obtain in large quantities and are hard to purify from culture filtrates without genetic manipulations.

### Phenotypic and genomic plasticities

Metabolic profilings showed *N. cyriacigeorgica* GUH-2 abilities at growing on a variety of substrates. In most cases, the CDSs involved in transport and catabolism of these substrates were found in the genome. However, some metabolic activities inferred from the genome could not be confirmed by growth tests e. g. the growth on L-glycine, L-lysine, and L-proline. Interestingly, CDSs involved in L-lysine and L-proline catabolism were detected outside RGP but showed a codon adaption index (CAI) below the average (data not shown). This codon bias could be related to the low expression of these CDSs, and might be the consequence of a recent acquisition. An overexpression of these genes by genetic manipulations of *N. cyriacigeorgica* GUH-2 would be needed to test this hypothesis. Low CAI was also observed for CDSs identified when *in vivo* activity was assessed. In this case the presence of multiple CDS (with both high and low CAI values) assigned to this function probably allowed a sufficient gene expression level to observe the expected phenotype.

A high turnover of RGP appears to occur among *N. cyriacigeorgica. N. cyriacigeorgica* GUH-2 closest RGP profile among a collection of 83 strains showed the absence of 7 RGP and the conservation of 15 ones. RGP profilings thus suggest a good *N. cyriacigeorgica* competence towards DNA acquisition but the mechanisms involved remain to be determined. RGP-Cy8 appeared to have been recently acquired by *N. cyriacigeorgica* GUH-2, and was found to harbor all the trademarks of mobile genomic islands. This RGP could be a good candidate for the design of a DNA cloning vector [57]. No RGP was found related to prophage-like elements. This is different from the situation observed in corynebacterial and mycobacterial pathogenic genomes, where such prophage-like DNA contains virulence genes [28].

A high turnover of IS elements was also observed among *Nocardia*. However, while several IS were found among the *N. cyriacigeorgica* (n = 16) and *N. farcinica* (n = 26) genomes in this work, previous studies had only identified two IS among the *Nocardia*: ISNfa1 (*N. farcinica*) [41] and IS204 (*N. mexicana*) [58]. These low numbers suggested *Nocardia* strains to have a poor propensity at acquiring exogenous DNA which are often acting as IS shuttles. Here, we clearly demonstrate the opposite. The *Nocardia* genomes were found to be rich in IS elements and diversity. IS of eight families were recorded suggesting a high genomic tolerance towards these elements and a frequent acquisition by these Actinobacteria. However, about 45% of the observed IS did not show IR and DR. A lack of such sequences could be indicative of a loss of transposition autonomy or of selective pressures leading to their fixation at a particular site because of functional benefits. This would need to be further investigated. IS were found involved in the emergence of pathogenic clones by reducing genome size [59]. There are also some reports showing a good match between the presence of a particular IS and infra-specific diversifications e.g. [60]. Distribution analysis also showed species-specific IS elements among the *Nocardia* e.g. ISNcy2 and ISNfa2 being restricted to *N. cyriacigeorgica* and *N. farcinica,* respectively. Furthermore, ISNcy3 was restricted to the *N. cyriacigeorgica* GUH-2 complex, and ISNfa5 of two particular clones of *N. farcinica*. These data support the hypothesis of a good match between IS acquisitions and infra-specific diversifications. Interestingly, distribution analysis indicated similar RGP/IS repartition profiles among *N. cyriacigeorgica* strains. These profiles divided *N. cyriacigeorgica* into two clusters: (1) those with patterns similar to strain GUH-2, and (2) those with patterns similar to the type strain. These similarities in the evolutionary patterns of these elements suggest a strong association. RGP could have been the genetic shuttles for some of these IS elements. In fact, even though most IS elements were found distributed over the genome without particular insertion site preferences, some e.g. ISNcy5, ISNcy2-b, ISNCy4, & ISNcy1-a4, were found harbored by RGP. For example, ISNcy2-b could have been acquired with RGP-Cy13. This IS is significantly different from the other ISNcy2-related copies found in the *N. cyriacigeorgica* GUH-2 genome, and has DNA signatures only detected on the copy found on this RGP. Interestingly, a division of *N. cyriacigeorgica* into two phylogenetic clusters was inferred by McTaggart *et al.*[13]. However, the strain collection of this latter study was different from the one of this work. It would be interesting to apply McTaggart *et al. *[13] approach to see if the IS/GI sub-groups would match such phylogenetic clusters. These analyses would add further support for the role of IS/GI elements in bacterial diversification and speciation. No relation between the habitat nor the geographical origin of the strains used in our study and the observed RGP/IS patterns could be inferred.

It is noteworthy that ISNcy2 was found similar to IS987 of *M. tuberculosis* and *M. bovis*, and was not detected in other sequenced bacterial genomes. In order to relate IS divergences to the evolution of Actinobacteria, the two orthologous IS alleles named ISNfa14 and ISNcy8, found in *N. farcinica* and *N. cyriacigeorgica,* respectively, were used as molecular clocks. These IS have 86% identity. Using this value, one can consider ISNcy2 and IS*987* diversification (75% identity) to be more ancient. PCR screenings showed ISNcy2 to be broadly distributed among *N. cyriacigeorgica* whereas it was totally absent from *N. farcinica* and other *Nocardia* type strains. This would suggest an acquisition by *N. cyriacigeorgica* at the moment of its differentiation.

### Outdoor-related functional ISues

Although a significant number of virulence genes were identified, with some being harbored by RGP, the main drivers of *N. cyriacigeorgica* evolution appear to be related to its environmental cycle outside mammalian hosts. Nocardioses are without a doubt of environmental origin, and infections are mainly the consequence of exposure to soil or water sources of these opportunistic pathogens. Indeed, several characteristics of the *N. cyriacigeorgica* GUH-2 genome reflect the environmental origin of this species. For instance, *N. cyriacigeorgica* genome size is in the range of what is expected for saprophytic bacteria (from 6 Mb to 11 Mb). This higher size range is related to the selection of CDS likely improving metabolic potentials and regulatory processes, and allowing growth under a wider range of environmental constraints [61]. These observations were further supported by COG analyses which showed *Nocardia* genomes to share a configuration similar to the ones observed among non-pathogenic Actinobacteria. Interestingly, both *Nocardia* genomes have a higher proportion of K and T COG CDS. These COGs encode DNA and proteins with regulatory functions, suggesting an ongoing evolution towards regulatory fine tunings of their genetic potentials that likely lead to expression patterns favoring growth under more diverse conditions.

## Conclusions

On one hand, primary pathogens are subjected to evolutionary forces driven by the host defense responses which can lead to a specialization for certain hosts and a genome size reduction as observed for *B. mallei*[62]. On the other hand, opportunistic pathogens are exposed to a multitude of environmental constraints that can favor an increased tolerance towards the acquisition of foreign DNA and the selection of novel metabolic properties. The *N. cyriacigeorgica* GUH-2 genome is a clear reflection of these latter trends. The *N. cyriacigeorgica* GUH-2 genome shows a great plasticity as shown from its RGP and IS patterns, which are strain-specific and appeared to have recently evolved. In fact, this genome appears to be undergoing important genetic rearrangements, and, most surprisingly, to frequently acquire novel DNA fragments. So far, *Nocardia* spp. were thought to have a low mating frequency, not acquiring much novel DNA from their neighbors. This work shows a completely opposite trend. *N. cyriacigeorgica* GUH-2 is clearly competent towards DNA or sexually active. This property can favor the gain of novel functions, and lead to major changes in niche preference from one strain to another such as differences in the colonization of certain human tISues.

## Methods

### Mice experimentations

Female BALB/c mice (pathogen-free) of 18-20 g (approximately 8 weeks old) were maintained by the “Institut Claude Bourgelat” (VetAgroSup, Marcy l’Etoile, France) in accordance with protocols approved by the board of ethics for animal experimentations.

*N. cyriacigeorgica* GUH-2 strain was grown in brain-heart infusion broth (BHI-P) to mid-log phase at 37°C with mild rotational agitation (150 rpm). The broth was centrifuged at low speed (55 *g*) to pellet bacterial clumps, and cell concentration was adjusted at approximately 3.5 × 10^6^ CFU/ml. A 0.1 ml of this suspension was injected intravenously (IV) through the lateral tail vein into each mouse, as described in details by Kohbata and Beaman [18]. Each mouse received approximately 3.5 × 10^5^ CFU.

### Genome sequencing and assembly

The genome sequence of *N. cyriacigeorgica* GUH-2 is publicly available at http://www.genoscope.cns.fr/agc/mage[63]. Sequences and annotations data have been deposited at the EMBL database (http://www.ebi.ac.uk/ena/) and given the accession number FO082843.1.

### Genome annotation and analysis

#### Synteny group computation

Sequence data for comparative analyses were obtained from the NCBI database (RefSeq section). Putative orthologies were defined as gene pairs satisfying either the BBH criterion or an alignment threshold (at least 40% sequence identity over at least 80% of the length of the smallest protein) [64]. These relationships were subsequently used to search for synteny groups (*i.e.* conservation of the chromosomal co-localization between pairs of orthologous genes from different genomes) among several bacterial genomes using an algorithm based on an exact graph-theoretical approach [65]. These results were used to draw a LinePlot using the MaGe MicroScope platform (https://www.genoscope.cns.fr/agc/microscope/home/index.php).

#### Detection of regions of genomic plasticity

The *RGPfinder* tool of the MicroScope annotation platform was used to identify Regions of Genomic Plasticity (RGP) in the whole genome sequence of *N. cyriacigeorgica* by using the *N. farcinica* sequence as a reference. RGP are defined as regions of at least 5 kb that are mISing in at least one of the genomes that are compared. This definition makes no assumption about the evolutionary origin or genetic basis of these variable chromosomal segments. *RGPfinder* searches for synteny breaks between a target genome and a set of closely related bacteria (generally other strains). It also provides information about composition abnormalities (%G + C deviation, Codon Adaptation Index) of these regions, and of their flanking sequences such as tRNA genes, IS and repeats, which are common features of RGP. Moreover, the tool integrates the results of Alien Hunter [66] and SIRGP-HMM [67], two methods that analyses compositional biases to detect atypical sequences (*i.e*. sequences potentially acquired by horizontal gene transfer). The whole genome of the *N. cyriacigeorgica* and *N. farcinica* were also aligned using ACT, a program for comparing two or more DNA sequences [68]. A region of five or more CDS and of more than 5 kb not retrieved in *N. farcinica* was also considered a RGP.

### Global comparative study

We compared genomes of *A. mediterranei* (CP002896.1), *C. diphtheriae* (BX248353), *C. glutamicum* (BA000036), *M. tuberculosis* (AL123456), *M. smegmatis* (CP000480), *N. farcinica* (AP006618), *N. cyriacigeorgica* (FO082843)*, R. equi* (ADNW00000000) and *R. jostii* (CP000431) using various graphic tools implemented on the MaGe Microscope platform and ACT. The phylogenetic tree of life of the Actinobacteria was built from a MLSA data set on the basis of [13]. We used Clusters of Orthologous Groups (COGs) automatic annotation and correspondence analysis (CA) with R software (http://www.R-project.org) [69] to graphically infer global trends between the above genomes. *Nocardia* IS families were identified using BLAST analyses against an IS database at https://www-is.biotoul.fr/.

Deleted CDS from the *N. cyriacigeorgica* GUH-2 genome were identified by searching CDS in common with *N. farcinica*, *R. equi* and *R. jostii* and looking for those absent from *N. cyriacigeorgica* genome using the Phyloprofiles exploration tool of MaGe. Only CDS presenting more than 40% amino-acid identities over 80% of the length of the shortest sequence were considered. Duplicated genes were detected in the *N. cyriacigeorgica* GUH-2 genome with the same tool, *N. cyriacigeorgica* genome was compared against itself and CDS presenting more than 70% predicted amino-acid identities were selected.

### Distribution of IS and selected RGP among a set of *Nocardia* strains

Rapid DNA extractions were performed on the suspension of 10 to 20 colonies of *Nocardia* cells in 100 μL of sterile water. The mixture was heated to 55°C for 15 min, and 5 units of achromopeptidase (Wako chemicals, Richmond VA) were added before incubating the suspension at 70°C for 15 min. Cells were centrifuged and the supernatant containing DNA was kept for further analyses. PCR screenings were designed to investigate the distribution of all selected RGP reported in this work, and for a set of selected IS elements. Primers were defined using primerselect (DNASTAR), and are indicated in the Additional file 12. All PCR reactions were performed in a final volume of 25 μL, containing 2.5 μL of each primers at 10 μM, 2.5 μL of 10X PCR buffer, 0.75 μL of MgCl_2_ 50 mM, 0.25 μL of 10 mM DNTPs, 5% DMSO w.vol^-1^, and 1 μL of the extracted DNA solution. PCR cycle is: 95°C 300 sec, 95°C 30 sec, annealing temperature depending on primers used, 72°C from 30 sec to 90 sec (× 35) and 72°C 300 sec. PCR products were visualized by electrophoresis using 1% agarose gels, and staining with ethidium bromide.

### Identification of the secretome

Protein secretion in Gram-positive bacteria occurs mainly through general secretory (Sec) and twin arginine translocation (Tat) pathways, and to a lesser extent by ABC (ATP-binding cassette) type transporters. There are also minor pathways such as the Early Secreted Antigen Target (ESAT-6) machinery described in *Mycobacterium*[70,71]. The secretome was analyzed for pathogenic and non pathogenic Actinobacteria using SignalP and TMhmm [72] as well as PSORTb [73]. These bioinformatics tools can detect trans-membrane domains [74]. None of these tools is error-free but their combined use yields a set of proteins that were previously shown, in most cases, to be secreted proteins [72,75].

### Metabolic profiling

*N. cyriacigeorgica* GUH-2 strain was grown for three days at 37°C on Middlebrook 7H10 (supplemented with 0.5% glycerol and 1% Middlebrook OADC enrichment) agar plates. *N. cyriacigeorgica* GUH-2 was inoculated to 20 mL of IF-0a GN/GP (Biolog Inc, Hayward CA, USA) and homogenized to obtain a 81% transmittance solution, free of bacterial clumps. This suspension (880 μL) was added to 10 mL of IF-0a GN/GP (Biolog Inc) supplemented with 1 mL of solution specific of each Omnilog plate. Dye mix (120 μL) F or H (depending on the Omnilog plate use) was added. This mix (100 μL) was added to each well of the selected Omnilog plates, and the plates were incubated. Cellular respiration was measured by monitoring formation of dark blue tetrazolium crystals over a 72 h time period.

## Abbreviations

CDS: Coding sequence; CMN: Corynebacteria, mycobacteria, and nocardia; COG: Cluster of orthologous groups; RGP: Region of genomic plasticity; IS: Insertion sequence; IR: Inverted DNA repeats; DR: Direct DNA repeats; NRPS: Non ribosomal peptide synthetase; PKS: Polyketide synthase; CAI: Codon adaptation index; MY: Million years

## Competing interests

The authors declare that they have no competing interests.

## Authors’ contributions

AZ, PP, AG, PB, YR, BC, and DB designed the study. AZ, PP, VRN, SR, DB and PBe performed the experiments. AZ, PBe, PB, PN, DR, DV, VB, SM, BC and DB analyzed the data sets. AZ, DB and BC drafted the manuscript; AZ, PP, PN, BC, and DB revised the manuscript and provided critical comments. All authors approved the final version of the manuscript.

## Supplementary Material

Additional file 1 **Virulence-related CDS found in the *N. cyriacigeorgica *GUH-2 genome.** Click here for file

Additional file 2 ***N. cyriacigeorgica *GUH-2 CDS occurrence and proportion in the CMN pangenome per COG.** Proportion of *N. cyriacygeorgica* CDS per COG was compared with the ones of the CMN pangenome by using the following formula: number of *N. cyriacygeorgica* CDS in a COG of the CMN pangenome divided by the total number of *N. cyriacygeorgica* CDS in this COG. Click here for file

Additional file 3 **Analysis of duplicated (threshold of 70% identity), lost (threshold of 40% identity), and RGP CDS of *N. cyriacigeorgica *GUH-2.** Deleted CDS from the *N. cyriacigeorgica* GUH-2 genome were identified by searching CDS in common with *N. farcinica*, *R. equi* and *R. jostii* and looking for those absent from *N. cyriacigeorgica* genome using the Phyloprofiles exploration tool of MaGe. Only CDS presenting more than 40% amino-acid identities over 80% of the length of the shortest sequence were considered. Click here for file

Additional file 4 Number of COGs and their relative proportion per species computed from nine Actinobacterial genomes. Click here for file

Additional file 5 **Correspondence Analysis of domains involved in transcription and retrieved in Am (*A. mediterranei*), Cd (*C. diphtheria*), Cg (*C. glutamicum*), Mt (*M. tuberculosis*), Ms (*M. smegmatis*), Nc (*N. cyriacigeorgica*), Nf (*N. farcinica*), Re (*R. equi*) and Rj (*R. jostii*).** Pathogenic Actinobacteria are represented in red, the non-pathogenic or saprophytic ones are in blue and *Nocardia* strains are in orange. Arrows show different proportions of COGs between pathogenic and non-pathogenic bacteria in the same genera. Transcription domains are as follow: ab (AbrB), ac (AraC), ar (ArsR), as (AsnC), cr (Crp), dr (DeoR), fu (Fur), gr (GntR), hr (HxlR), ir (IclR), li (LacI), lr (LuxR), ly (LysR), ma (MarR), me (MerR), mo (MoxR), nr (NrdR), pr (PadR), r2 (Rrf2), tf (CarD/TRCF), tr (TetR) and wb (WhiB). Click here for file

Additional file 6 Number of CDS containing putative domains involved in transcription and their relative proportion per species as computed from nine Actinobacterial genomes. Click here for file

Additional file 7 **General features of IS identified in the *N. cyriacigeorgica *and *N. farcinica *genomes.** Click here for file

Additional file 8 **Number of CDS associated with the secretome of Actinobacteria, and their relative proportion per species (Nc: *N. cyriacigeorgica*, Nf: *N. farcinica*, Rj: *R. jostii*, Re: *R. equi*, Ms:*M. smegmatis*, Mt: *M. tuberculosis*, Cg: *C. glutamicum*, Cd: *C. diphtheria*, Am: *A. mediterranei*).** Each CDS was associated to a COG. Click here for file

Additional file 9 PCR primers used in this study for RGP and IS distribution analyses. Click here for file

Additional file 10 **Regions of genomic plasticity (Cy code) detected by PCR in 83  *N. cyriacigeorgica *strains.** N. farcinica DNA was used as a control. Click here for file

Additional file 11 **Distribution of selected IS (ISNCy) among *N. cyriacigeorgica *and *N. farcinica *strains (PCR screenings); positive results are in grey.** Click here for file

Additional file 12 **OmniLog® metabolic profilings of *N. cyriacigeorgica *GUH-2 cells.** Functional predictions made from genome DNA sequence analyses are indicated. Utilization of various carbon and nitrogen sources, and osmolite resistances were investigated. Differences in the data sets are indicated in grey. Click here for file
