# Supplementary material for: The Nocardia cyriacigeorgica GUH-2 genome shows ongoing adaptation of an environmental Actinobacteria to a pathogen’s lifestyle
Source: BMC Genomics. 2013 Apr 27;14:286. doi: 10.1186/1471-2164-14-286 (PMC3751702; doi:10.1186/1471-2164-14-286)
Supplement: Additional file 1 — Virulence-related CDS found in the N. cyriacigeorgica GUH-2 genome. [file 1471-2164-14-286-S1.pdf]

Gene Family	Gene name
mce family protein	nocyr_0547, nocyr_0548, nocyr_0549, nocyr_0550, nocyr_0551, nocyr_0552, nocyr_0553, nocyr_0554, nocyr_0555,<br>nocyr_1218, nocyr_1219, nocyr_1220, nocyr_1221, nocyr_1222, nocyr_1223,<br>nocyr_1230, nocyr_1231, nocyr_1232, nocyr_1233, nocyr_1234, nocyr_1235, nocyr_1236, nocyr_1237,<br>nocyr_1542, nocyr_1543, nocyr_1544, nocyr_1545, nocyr_1546, nocyr_1548,<br>nocyr_4380,<br>nocyr_4829, nocyr_4830, nocyr_4831, nocyr_4832, nocyr_4833, nocyr_4834, nocyr_4835, nocyr_4836,<br>nocyr_4888, nocyr_4889, nocyr_4890, nocyr_4891, nocyr_4892, nocyr_4893, nocyr_4894, nocyr_4895,<br>nocyr_5135, nocyr_5136,<br>nocyr_5524, nocyr_5525, nocyr_5526, nocyr_5527, nocyr_5528, nocyr_5529, nocyr_5530, nocyr_5531
antigen 85 protein	nocyr_0138, nocyr_0139,<br>nocyr_1255, nocyr_1792, nocyr_4128
ESAT-6 protein	nocyr_0850, nocyr_0851, nocyr_0891
lipoprotein	nocyr_0332, nocyr_0442, nocyr_1182, nocyr_1280, nocyr_1594, nocyr_1858, nocyr_1895,<br>nocyr_2023, nocyr_2288, nocyr_2563, nocyr_2787, nocyr_3283, nocyr_3358, nocyr_4333,<br>nocyr_4547, nocyr_4621, nocyr_4622, nocyr_4633, nocyr_4864
EIS protein	nocyr_0655
PhoP/PhoR system	nocyr_0566, nocyr_0567
mycosin	nocyr_1375
hemolysin	nocyr_2189, nocyr_4593
lipid II flippase	nocyr_5555
acyl transferase	nocyr_5047
isocitrate lyase	nocyr_5051
invasion associated protein	nocyr_1837, nocyr_4599, nocyr_5488
endoribonuclease mazF	nocyr_5508
dihydroxybenzoate	nocyr_0626, nocyr_4112, nocyr_4387
SOD	nocyr_0085, nocyr_5123
Catalase	nocyr_0300, nocyr_2216, nocyr_2744
