# Supplementary material for: The Nocardia cyriacigeorgica GUH-2 genome shows ongoing adaptation of an environmental Actinobacteria to a pathogen’s lifestyle
Source: BMC Genomics. 2013 Apr 27;14:286. doi: 10.1186/1471-2164-14-286 (PMC3751702; doi:10.1186/1471-2164-14-286)
Supplement: Additional file 2 — N. cyriacigeorgica GUH-2 CDS occurrence and proportion in the CMN pangenome per COG. Proportion of N. cyriacygeorgica CDS per COG was compared with the ones of the CMN pangenome by using the following formula: number of N. cyriacygeorgica CDS in a COG of the CMN pangenome divided by the total number of N. cyriacygeorgica CDS in this COG. [file 1471-2164-14-286-S2.pdf]

COG Process	COG ID	Description	*N. cyriacigeorgica* CDS in CMN pangenome	
			CDS number	CDS proportion
CELLULAR PROCESSES AND SIGNALING	D	Cell cycle control, cell division, chromosome partitioning	17	32.7%
	M	Cell wall/membrane/envelope biogenesis	54	29.0%
	N	Cell motility	10	13.3%
	O	Posttranslational modification, protein turnover, chaperones	48	31.2%
	T	Signal transduction mechanisms	43	13.9%
	U	Intracellular trafficking, secretion, and vesicular transport	15	32.6%
	V	Defense mechanisms	20	14.2%
	W	Extracellular structures	0	0.0%
INFORMATION STORAGE AND PROCESSING	A	RNA processing and modification	1	100.0%
	B	Chromatin structure and dynamics	1	50.0%
	J	Translation, ribosomal structure and biogenesis	135	65.5%
	K	Transcription	75	11.8%
	L	Replication, recombination and repair	72	29.4%
METABOLISM	C	Energy production and conversion	49	14.4%
	E	Amino acid transport and metabolism	127	19.0%
	F	Nucleotide transport and metabolism	46	40.4%
	G	Carbohydrate transport and metabolism	54	14.3%
	H	Coenzyme transport and metabolism	74	36.6%
	I	Lipid transport and metabolism	43	11.%
	P	Inorganic ion transport and metabolism	50	11.1%
	Q	Secondary metabolites biosynthesis, transport and catabolism	18	4.6%
POORLY CHARACTERIZED	R	General function prediction only	98	10.0%
	S	Function unknown	38	14.0%

	(all CDS)	
	CDS number	% of genome CDS
	52	0.9%
	186	3.4%
	75	1.4%
	154	2.8%
	310	5.7%
	46	0.8%
	141	2.6%
	1	0.0%
	1	0.0%
	2	0.0%
	206	3.8%
	636	11.6%
	245	4.5%
	340	6.2%
	668	12.2%
	114	2.1%
	378	6.9%
	202	3.7%
	384	7.0%
	449	8.2%
	391	7.2%
	978	17.9%
	272	5.0%
