# Supplementary material for: The Nocardia cyriacigeorgica GUH-2 genome shows ongoing adaptation of an environmental Actinobacteria to a pathogen’s lifestyle
Source: BMC Genomics. 2013 Apr 27;14:286. doi: 10.1186/1471-2164-14-286 (PMC3751702; doi:10.1186/1471-2164-14-286)
Supplement: Additional file 3 — Analysis of duplicated (threshold of 70% identity), lost (threshold of 40% identity), and RGP CDS of N. cyriacigeorgica GUH-2. Deleted CDS from the N. cyriacigeorgica GUH-2 genome were identified by searching CDS in common with N. farcinica, R. equi and R. jostii and looking for those absent from N. cyriacigeorgica genome using the Phyloprofiles exploration tool of MaGe. Only CDS presenting more than 40% amino-acid identities over 80% of the length of the shortest sequence were considered. [file 1471-2164-14-286-S3.pdf]

| *N. cyriacigeorgica* | Nb. CDS | Not retrived in *N. farcinica* | COGs mostly represented |
|---|---|---|---|
| RGP | 1563 | 515 | R, K, E, Q |
| Duplicated Genes | 161 | 59 | Q, C, I |
| Lost genes (*N. farcinica, R. jostii, R. equi* ) | 193 | 0 | E, R, Q, K |

| *N. cyriacigeorgica* | Nb. CDS | Not retrived in *N. farcinica* | COGs mostly represented |
|---|---|---|---|
| RGP | 1563 | 515 | R, K, E, Q |
| Duplicated Genes | 161 | 59 | Q, C, I |
| Lost genes (*N. farcinica, R. jostii, R. equi* ) | 193 | 0 | E, R, Q, K |
