# Supplementary material for: The Nocardia cyriacigeorgica GUH-2 genome shows ongoing adaptation of an environmental Actinobacteria to a pathogen’s lifestyle
Source: BMC Genomics. 2013 Apr 27;14:286. doi: 10.1186/1471-2164-14-286 (PMC3751702; doi:10.1186/1471-2164-14-286)
Supplement: Additional file 4 — Number of COGs and their relative proportion per species computed from nine Actinobacterial genomes. [file 1471-2164-14-286-S4.pdf]

COG ID	Occurrence, (%)								
	N. cyriacigeorgica	*N. farcinica*	*R. jostii*	*R. equi*	*M. smegmatis*	*M. tuberculosis*	*C. glutamicum*	*C. diphtheriae*	*A. mediterranei*
A	1 (0.02)	1 (0.02)	1 (0.01)	1 (0.03)	1 (0.02)	1 (0.03)	1 (0.04)	1 (0.06)	1 (0.01)
B	2 (0.05)	3 (0.07)	1 (0.01)	1 (0.03)	2 (0.04)	2 (0.07)	0 (0.00)	0 (0.00)	2 (0.02)
C	340 (8.47)	379 (8.91)	741 (10.96)	352 (9.26)	614 (11.42)	281 (9.51)	173 (7.49)	129 (7.17)	644 (6.45)
D	52 (1.29)	51 (1.20)	69 (1.02)	45 (1.18)	51 (0.95)	46 (1.56)	34 (1.47)	28 (1.56)	71 (0.71)
E	668 (16.63)	732 (17.21)	1282 (18.96)	741 (19.49)	1051 (19.55)	481 (16.27)	471 (20.39)	335 (18.63)	1246 (12.48)
F	114 (2.84)	127 (2.99)	197 (2.91)	124 (3.26)	171 (3.18)	105 (3.55)	95 (4.11)	84 (4.67)	184 (1.84)
G	378 (9.41)	367 (8.63)	714 (10.56)	366 (9.63)	615 (11.44)	237 (8.02)	290 (12.55)	180 (10.01)	937 (9.38)
H	202 (5.03)	212 (4.98)	290 (4.29)	175 (4.60)	234 (4.35)	157 (5.31)	129 (5.58)	114 (6.34)	313 (3.13)
I	384 (9.56)	408 (9.59)	797 (11.79)	435 (11.44)	607 (11.29)	314 (10.62)	98 (4.24)	77 (4.28)	587 (5.88)
J	206 (5.13)	237 (5.57)	252 (3.73)	205 (5.39)	216 (4.02)	178 (6.02)	182 (7.88)	173 (9.62)	265 (2.65)
K	636 (15.84)	657 (15.44)	904 (13.37)	484 (12.73)	674 (12.54)	254 (8.59)	232 (10.04)	158 (8.79)	1246 (12.48)
L	245 (6.10)	280 (6.58)	479 (7.08)	182 (4.79)	297 (5.53)	250 (8.46	201 (8.70)	202 (11.23)	343 (3.43)
M	186 (4.63)	180 (4.23)	248 (3.67)	162 (4.26)	208 (3.87)	139 (4.70)	122 (5.28)	93 (5.17)	359 (3.59)
N	75 (1.87)	81 (1.90)	92 (1.36)	63 (1.66)	85 (1.58)	52 (1.76)	30 (1.30)	42 (2.34)	114 (1.14)
O	154 (3.83)	171 (4.02)	225 (3.33)	484 (12.73)	175 (3.26)	156 (5.28)	105 (4.55)	158 (8.79)	256 (2.56)
P	449 (11.18)	496 (11.66)	817 (12.08)	533 (14.02)	673 (12.52)	279 (9.44)	359 (15.54)	239 (13.29)	740 (7.41)
Q	391 (9.74)	388 (9.12)	691 (10.22)	343 (9.02)	578 (10.75)	361 (12.21)	81 (3.51)	52 (2.89)	637 (6.38)
R	978 (24.35)	1000 (23.51)	1676 (24.79)	940 (24.73)	1325 (24.65)	642 (21.72)	494 (21.39)	299 (16.63)	1761 (17.63)
S	272 (6.77)	270 (6.35)	425 (6.29)	252 (6.63)	325 (6.05)	206 (6.97)	191 (8.27)	136 (7.56)	434 (4.35)
T	310 (7.72)	297 (6.98)	419 (6.20)	213 (5.60)	300 (5.58)	183 (6.19)	111 (4.81)	105 (5.84)	570 (5.71)
U	46 (1.15)	47 (1.10)	50 (0.74)	51 (1.34)	45 (0.84)	52 (1.76)	34 (1.47)	33 (1.84)	79 (0.79)
V	141 (3.51)	155 (3.64)	168 (2.48)	120 (3.16)	144 (2.68)	76 (2.57)	103 (4.46)	85 (4.73)	221 (2.21)
W	1 (0.02)	0 (0.00)	0 (0.00)	1 (0.03)	1 (0.02)	0 (0.00)	0 (0.00)	0 (0.00)	4 (0.04)
Tot:	4016	4254	6761	3801	5375	2956	2310	1798	7106
