# Supplementary figures and images for: The Nocardia cyriacigeorgica GUH-2 genome shows ongoing adaptation of an environmental Actinobacteria to a pathogen’s lifestyle
Source: BMC Genomics. 2013 Apr 27;14:286. doi: 10.1186/1471-2164-14-286 (PMC3751702; doi:10.1186/1471-2164-14-286)

# Transcription domains

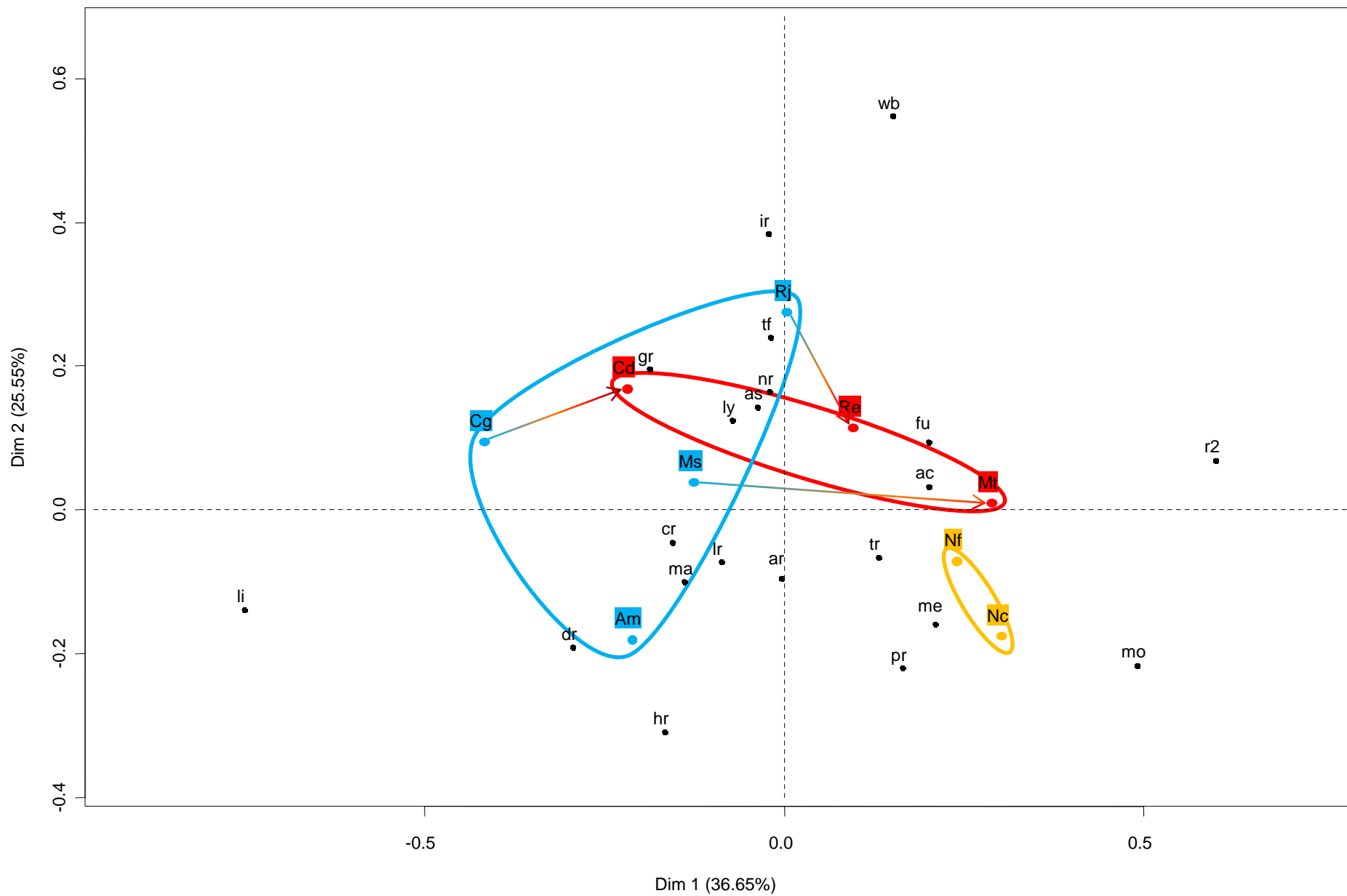

Supplement: Additional file 5 — Correspondence Analysis of domains involved in transcription and retrieved in Am (A. mediterranei), Cd (C. diphtheria), Cg (C. glutamicum), Mt (M. tuberculosis), Ms (M. smegmatis), Nc (N. cyriacigeorgica), Nf (N. farcinica), Re (R. equi) and Rj (R. jostii). Pathogenic Actinobacteria are represented in red, the non-pathogenic or saprophytic ones are in blue and Nocardia strains are in orange. Arrows show different proportions of COGs between pathogenic and non-pathogenic bacteria in the same genera. Transcription domains are as follow: ab (AbrB), ac (AraC), ar (ArsR), as (AsnC), cr (Crp), dr (DeoR), fu (Fur), gr (GntR), hr (HxlR), ir (IclR), li (LacI), lr (LuxR), ly (LysR), ma (MarR), me (MerR), mo (MoxR), nr (NrdR), pr (PadR), r2 (Rrf2), tf (CarD/TRCF), tr (TetR) and wb (WhiB). [file 1471-2164-14-286-S5.pdf]
