# Supplementary material for: The Nocardia cyriacigeorgica GUH-2 genome shows ongoing adaptation of an environmental Actinobacteria to a pathogen’s lifestyle
Source: BMC Genomics. 2013 Apr 27;14:286. doi: 10.1186/1471-2164-14-286 (PMC3751702; doi:10.1186/1471-2164-14-286)
Supplement: Additional file 6 — Number of CDS containing putative domains involved in transcription and their relative proportion per species as computed from nine Actinobacterial genomes. [file 1471-2164-14-286-S6.pdf]

| Transcription domains | Occurrence, (%) | | | | | | | | |
|---|---|---|---|---|---|---|---|---|---|
| | *N. cyriacigeorgica* | *N. farcinica* | *R. jostii* | *R. equi* | *M. smegmatis* | *M. tuberculosis* | *C. glutamicum* | *C. diphtheriae* | *A. mediterranei* |
| AbrB | 1 (0.25) | 0 0 | 0 0 | 0 0 | 0 0 | 2 (1.44) | 0 0 | 0 0 | 0 0 |
| AraC | 29 (7.34) | 36 (8.55) | 46 (7.03) | 19 (5.48) | 24 (4.95) | 7 (5.04) | 4 (3.57) | 1 (1.79) | 39 (4.66) |
| ArsR | 19 (4.81) | 23 (5.46) | 27 (4.13) | 11 (3.17) | 14 (2.89) | 12 (8.63) | 11 (9.82) | 2 (3.57) | 44 (5.26) |
| AsnC | 7 (1.77) | 7 (1.66) | 23 (3.52) | 9 (2.59) | 11 (2.27) | 5 (3.6) | 2 (1.79) | 0 0 | 22 (2.63) |
| Crp | 1 (0.25) | 2 (0.48) | 2 (0.31) | 1 (0.29) | 2 (0.41) | 3 (2.16) | 2 (1.79) | 1 (1.79) | 5 (0.6) |
| DeoR | 6 (1.52) | 6 (1.43) | 6 (0.92) | 5 (1.44) | 8 (1.65) | 0 0 | 4 (3.57) | 2 (3.57) | 19 (2.27) |
| Fur | 3 (0.76) | 3 (0.71) | 3 (0.46) | 2 (0.58) | 4 (0.82) | 2 (1.44) | 1 (0.89) | 1 (1.79) | 2 (0.24) |
| GntR | 19 (4.81) | 26 (6.18) | 72 (11.01) | 27 (7.78) | 65 (13.4) | 8 (5.76) | 12 (10.71) | 8 (14.29) | 66 (7.89) |
| HxlR | 7 (1.77) | 10 (2.38) | 8 (1.22) | 4 (1.15) | 11 (2.27) | 2 (1.44) | 2 (1.79) | 0 0 | 27 (3.23) |
| IclR | 8 (2.03) | 19 (4.51) | 52 (7.95) | 32 (9.22) | 22 (4.54) | 4 (2.88) | 7 (6.25) | 2 (3.57) | 29 (3.46) |
| LacI | 0 0 | 3 (0.71) | 18 (2.75) | 4 (1.15) | 25 (5.15) | 1 (0.72) | 10 (8.93) | 3 (5.36) | 56 (6.69) |
| LuxR | 38 (9.62) | 29 (6.89) | 59 (9.02) | 31 (8.93) | 37 (7.63) | 8 (5.76) | 8 (7.14) | 6 (10.71) | 96 (11.47) |
| LysR | 22 (5.57) | 24 (5.7) | 49 (7.49) | 23 (6.63) | 40 (8.25) | 5 (3.6) | 10 (8.93) | 3 (5.36) | 46 (5.5) |
| MarR | 23 (5.82) | 23 (5.46) | 37 (5.66) | 27 (7.78) | 27 (5.57) | 7 (5.04) | 9 (8.04) | 4 (7.14) | 75 (8.96) |
| MerR | 30 (7.59) | 22 (5.23) | 20 (3.06) | 14 (4.03) | 13 (2.68) | 4 (2.88) | 5 (4.46) | 4 (7.14) | 30 (3.58) |
| MoxR | 4 (1.01) | 3 (0.71) | 2 (0.31) | 1 (0.29) | 3 (0.62) | 3 (2.16) | 0 0 | 0 0 | 3 (0.36) |
| NrdR | 1 (0.25) | 1 (0.24) | 1 (0.15) | 1 (0.29) | 1 (0.21) | 1 (0.72) | 1 (0.89) | 1 (1.79) | 1 (0.12) |
| PadR | 13 (3.29) | 13 (3.09) | 10 (1.53) | 6 (1.73) | 5 (1.03) | 3 (2.16) | 3 (2.68) | 1 (1.79) | 19 (2.27) |
| Rrf2 | 3 (0.76) | 3 (0.71) | 3 (0.46) | 2 (0.58) | 2 (0.41) | 2 (1.44) | 0 0 | 0 0 | 1 (0.12) |
| CarD/TRCF | 2 (0.51) | 2 (0.48) | 3 (0.46) | 2 (0.58) | 2 (0.41) | 2 (1.44) | 2 (1.79) | 2 (3.57) | 2 (0.24) |
| TetR | 151 (38.23) | 157 (37.29) | 179 (27.37) | 119 (34.29) | 163 (33.61) | 52 (37.41) | 15 (13.39) | 12 (21.43) | 248 (29.63) |
| WhiB | 8 (2.03) | 9 (2.14) | 34 (5.2) | 7 (2.02) | 6 (1.24) | 6 (4.32) | 4 (3.57) | 3 (5.36) | 7 (0.84) |
| Tot: | 395 | 421 | 654 | 347 | 485 | 139 | 112 | 56 | 837 |
