# Supplementary material for: The Nocardia cyriacigeorgica GUH-2 genome shows ongoing adaptation of an environmental Actinobacteria to a pathogen’s lifestyle
Source: BMC Genomics. 2013 Apr 27;14:286. doi: 10.1186/1471-2164-14-286 (PMC3751702; doi:10.1186/1471-2164-14-286)
Supplement: Additional file 7 — General features of IS identified in the N. cyriacigeorgica and N. farcinica genomes. [file 1471-2164-14-286-S7.pdf]

Souche	Cy1	Cy2	Cy3	Cy4	Cy5	Cy6	Cy7	Cy8	Cy9	Cy10	Cy11	Cy12	Cy13	Cy14	Cy15	Cy16	Cy17	Cy18	Cy19	Cy20	Cy21	Cy22
**GUH-2**																						
N7																						
04.91																						
09.17																						
N27																						
N32																						
976.																						
06.38																						
03.86																						
08.65																						
N19																						
N37																						
AZA																						
09.102																						
N20																						
05.80																						
06.143																						
EK																						
03.54																						
05.106																						
05.128																						
07.77																						
07.114																						
09.51																						
09.142																						
04.09																						
08.119																						
OK.AK																						
X																						
03.39																						
07.50																						
SU.HA																						
05.48																						
BA.US																						
03.50																						
04.12																						
05.19																						
06.78																						
06.90																						
07.38																						
09.15																						
GE																						
N3																						
N13																						
N16																						
N22																						
N35																						
04.100																						
05.69																						
06.106																						
07.186																						
08.15																						
08.210																						
09.124																						
09.131																						
07.184																						
N5																						
N6																						
N34																						
05.09																						
05.44																						
05.46																						
05.116																						
06.67																						
06.129																						
07.93																						
08.82																						
08.149																						
08.163																						
AKO.05																						
BE1.05																						
COB.05																						
YES.05																						
KUP.05																						
ISYU.1																						
ISYU.2																						
ME																						
CET.IM																						
1371.																						
08036177.																						
09.93																						
YES.HE																						
**FARC.10152**																						

GUH-2's strains complex

Type's strains complex

N. cyriacigeorgica

N. farcinica
