# Supplementary material for: The Nocardia cyriacigeorgica GUH-2 genome shows ongoing adaptation of an environmental Actinobacteria to a pathogen’s lifestyle
Source: BMC Genomics. 2013 Apr 27;14:286. doi: 10.1186/1471-2164-14-286 (PMC3751702; doi:10.1186/1471-2164-14-286)
Supplement: Additional file 8 — Number of CDS associated with the secretome of Actinobacteria, and their relative proportion per species (Nc: N. cyriacigeorgica, Nf: N. farcinica, Rj: R. jostii, Re: R. equi, Ms:M. smegmatis, Mt: M. tuberculosis, Cg: C. glutamicum, Cd: C. diphtheria, Am: A. mediterranei). Each CDS was associated to a COG. [file 1471-2164-14-286-S8.pdf]

	IS Name	Family - Group	IR left	IR right	Number of copies and DNA allelic % identity	DR	Length
N. cyriacigeorgica	ISNcy1-a1	IS256	GGGAGTGTCTAATTAACGGCGGATCT	AGATCAGCCGTTAACTGGACACACCC		CTTGGCGA	1400
	ISNcy1-a2	IS256	GGGAGTGTCTAATTAACGGCGGATCT	AGATCAGCCGTTAACTGGACACACCC	4 copies with 100 % identities	none	1384
	ISNcy1-a3	IS256	GGGAGTGTCTAATTAACGGCGGATCT	AGATCAGCCGTTAACTGGACACACCC		G	1386
	ISNcy1-a4 (fragment)	IS256	none	AGATCAGCCGTTAACTGGACACACCC		none	512
	ISNcy2-a1	IS3 - IS51	TGAATCCCCCCGGCGATTCCGGAGGC	GTCTCCGGACATGCCGGGGGGATTCA		C	1343
	ISNcy2-a2	IS3 - IS51	TGAATCCCCCCGGCGATTCCGGAGGC	GTCTCCGGACATGCCGGGGGGATTCA	3 copies from 82 to 100 % identities	CTCG	1349
	ISNcy2-b	IS3 - IS51	TGAATCCCCCGGCATGTCCGGAGGC	GTCTCCGGACTCACCGGGGCGATTCA		CCCG	1350
	ISNcy3	Tn3	GGGGTTCCGGTAGTAACCGCCGAAGAATCAACGGATATGGCGTAGC	GCTACGCCATATCCGTTGGTTTTTCGGCGGTTACTACCGGAACCCC		AG	2166
	ISNcy4	Tn3	CGGGGTCGCCGTAGTATCGGCCGATTTTCGGGCGGATATGGCGTA	TACGCCATATCCGCCCGAAATTCGGCCGATACTACGGCGACCCCG		ACC	6178
	ISNcy5	IS200/IS605 - IS608	none	none		none	375
	ISNcy6	IS200/IS605 - IS1341	none	none		none	1143
	ISNcy7	ISNCY	none	none		none	1005
	ISNcy8	IS3 - IS51	none	none		none	309
	ISNcy9	ISNCY	none	none		none	534
	ISNcy10	IS21	none	none		none	207
	ISNcy11	ISNCY	CGGCGAACATCGAAGTACACAAC	GTTCTGTAGCTCGGTACCTGCCG		none	906
N. farcinica	ISNfa1-a	IS481	TGTTGCGGGTCGTGACGTTGGTGACGCGGTGGCGGTGGTGAGCTGAGGGC	ACCAGCCAGCCGCCTCCACACCGGCGTCACCAACGTCACGGCCTCATACA	2 copies with 99 % identities	none	1112
	ISNfa1-b	IS481	TGTTGCGGGTCGTGACGTTGGTGACGCGGTGGCGGTGGTGAGCTGAGGGC	ACCAGCCAGCCGCCTCCACACCGGCGTCACCAACGTCACGGCCTCATACA		none	1113
	ISNfa2-a	IS5 - IS427	CTAGGCGGTGTCTCGAAGTAG	CTACTTCGAGACACCGCCTAG		none	1048
	ISNfa2-b	IS5 - IS427	CTAGGCAGTGTCTCGAAGTAG	CTACTTCGAGACACCGCCTAG		none	1048
	ISNfa2-c	IS5 - IS427	none	none		none	906
	ISNfa2-d	IS5 - IS427	CTAGGCGGTGTCTCGAAGTAG	CTACTTCGAGACACCGCCTAG	8 copies from 72 to 99 % identities	none	1048
	ISNfa2-e	IS5 - IS427	CTAGGCGGTGTCTCGAAGTAG	CTACTTCGAGACACCGCCTAG		none	1048
	ISNfa2-f	IS5 - IS427	CTAGGCAGTGTCTCGAAGTAG	CTACTTCGAGACACCGCCTAG		none	1048
	ISNfa2-g	IS5 - IS427	CCTAGGCGGTGTCTCGAAGTAG	CTACTTCGAGACACCGCCTAGG		none	1051
	ISNfa2-h	IS5 - IS427	CTAGGCAGTGTCTCGAAGTAG	CTACTTCGAGACACCGCCTAG		none	1048
	ISNfa3	IS5 - IS427	none	none		none	411
	ISNfa4	IS200/IS605	none	none		none	1221
	ISNfa5	IS200/IS605	none	none		none	1146
	ISNfa6	IS5 - IS427	none	none		none	321
	ISNfa7	IS481	none	none		none	1032
	ISNfa8	IS630	CCTAGTGCCGCG	CGCGGCACTAGG		none	1199
	ISNfa9	IS3 -IS3	none	none		none	366
	ISNfa10	IS3 - IS407	none	none		none	246
	ISNfa11	IS3 -IS3	none	none		none	255
	ISNfa12	IS3 -IS3	none	none		none	928
	ISNfa13	IS3 -IS3	none	none		none	351
	ISNfa14	IS3 - IS51	none	none		none	309
	ISNfa15	IS5 - IS427	none	none		none	1107
	ISNfap1-a	Tn3	GGGGTAGCCGTAGCATCCGCCGAAAATCAGGCGGATATGGCGT	ACGCCATATCCGCCCGATTTTCGGCAAATGCTACGGCTACCCC		none	7260
	ISNfap1-b (fragment)	Tn3	none	none	2 copies with 85 % identities	none	696
	ISNfap2	IS3 - IS51	none	none		none	234
