# Supplementary material for: The Nocardia cyriacigeorgica GUH-2 genome shows ongoing adaptation of an environmental Actinobacteria to a pathogen’s lifestyle
Source: BMC Genomics. 2013 Apr 27;14:286. doi: 10.1186/1471-2164-14-286 (PMC3751702; doi:10.1186/1471-2164-14-286)
Supplement: Additional file 9 — PCR primers used in this study for RGP and IS distribution analyses. [file 1471-2164-14-286-S9.pdf]

Group	Strain	ISNcy2	ISNcy3	ISNcy4	ISNfa1	ISNfa2	ISNfa5
N. cyriacigeorgica – GUH-2 complex	**GUH2**	■	■	■			
	N7	■					
	N27	■		■			
	03.86	■					
	04.91	■	■	■			
	06.38	■					
	09.17	■	■				
	976.	■	■				
	AZA	■	■				
	09.102	■	■				
N. cyriacigeorgica – Type strain complex	**DSM 44484**						
	N13						
	04.12	■					
	05.69	■					
	07.93	■					
	08.02	■					
	09.131	■					
	AKO	■					
	BE1						
	1351.					■	
N. farcinica	**10152.**			■	■	■	■
	**DSM 43665**			■	■	■	■
	07.48			■		■	
	08.148						
	08.150			■		■	
	08..166			■		■	
	08.106			■		■	
	09.27			■		■	
	09.45			■		■	
	09.53			■		■	
	4037.			■		■	
	5596.			■		■	
Nocardia type strains	*N. abcsessus*						
	N. asteroides						
	N. brasiliensis						
	N. carnea						
	N. nova						
	N. otitidiscaviarum	■					
	N. paucivaurans						
	N. mexicana						
	N. tranvalensis						
	N. vinacea						
