# Supplementary material for: The Nocardia cyriacigeorgica GUH-2 genome shows ongoing adaptation of an environmental Actinobacteria to a pathogen’s lifestyle
Source: BMC Genomics. 2013 Apr 27;14:286. doi: 10.1186/1471-2164-14-286 (PMC3751702; doi:10.1186/1471-2164-14-286)
Supplement: Additional file 10 — Regions of genomic plasticity (Cy code) detected by PCR in 83 N. cyriacigeorgica strains. N. farcinica DNA was used as a control. [file 1471-2164-14-286-S10.pdf]

COG process	Occurrence, (%)								
	N. cyriacigeorgica	*N. farcinica*	*R. jostii*	*R. equi*	*M. smegmatis*	*M. tuberculosis*	*C. glutamicum*	*C. diphtheriae*	*A. mediterranei*
SP[(1)]>0,5 TMh[(2)]<3	263 (4.80)	281 (4.70)	249 (3.16)	256 (5.00)	326 (4.38)	184 (4.13)	129 (4.12)	139 (5.58)	621 (6.22)
Cellular process and signaling	53 (20.15)	70 (24.91)	74 (29.72)	58 (22.66)	74 (22.70)	41 (22.28)	33 (25.58)	34 (24.46)	125 (20.13)
Information storage and processing	2 (0.76)	4 (1.42)	3 (1.20)	3 (1.17)	3 (0.92)	5 (2.72)	1 (0.78)	2 (1.44)	8 (1.29)
Metabolism	51 (19.39)	47 (16.73)	66 (26.51)	51 (19.92)	77 (23.62)	38 (20.65)	21 (16.28)	15 (10.79)	147 (23.67)
Poorly characterized	49 (18.63)	45 (16.01)	53 (21.29)	42 (16.41)	34 (10.43)	27 (14.67)	27 (20.93)	9 (6.47)	66 (10.63)
all COG	127 (48.29)	137 (48.75)	157 (63.05)	132 (51.56)	158 (48.47)	92 (50.00)	70 (54.26)	57 (41.01)	298 (47.99)
Hypothetical	185 (70.34)	189 (67.26)	145 (58.23)	166 (64.84)	202 (61.96)	119 (64.67)	86 (66.67)	91 (65.47)	389 (62.64)
nb of CDS in genome	5477	5984	7872	5124	7449	4454	3128	2491	9988

[(1)]SP: Signal peptide, [(2)]TMh: transmembrane helix domain
