# Supplementary material for: The Nocardia cyriacigeorgica GUH-2 genome shows ongoing adaptation of an environmental Actinobacteria to a pathogen’s lifestyle
Source: BMC Genomics. 2013 Apr 27;14:286. doi: 10.1186/1471-2164-14-286 (PMC3751702; doi:10.1186/1471-2164-14-286)
Supplement: Additional file 11 — Distribution of selected IS (ISNCy) among N. cyriacigeorgica and N. farcinica strains (PCR screenings); positive results are in grey. [file 1471-2164-14-286-S11.pdf]

Group	Subgroup	Strain	ISNcy2	ISNcy3	ISNcy4	ISNfa1	ISNfa2	ISNfa5
N. cyriacigeorgica	GUH-2 complex	**GUH2**	■	■	■			
		N7	■					
		N27	■		■			
		03.86	■					
		04.91	■	■	■			
		06.38	■					
		09.17	■	■				
		976.	■	■				
		AZA	■	■				
		09.102	■	■				
	Type strain complex	**DSM 44484**						
		N13						
		04.12	■					
		05.69	■					
		07.93	■					
		08.02	■					
		09.131	■					
		AKO	■					
		BE1						
		1351.					■	
N. farcinica		**10152.**			■	■	■	■
		**DSM 43665**			■	■	■	■
		07.48			■		■	
		08.148						
		08.150			■		■	
		08..166			■		■	
		08.106			■		■	
		09.27			■		■	
		09.45			■		■	
		09.53			■		■	
		4037.			■		■	
		5596.			■		■	
Nocardia type strains		*N. abcsessus*						
		N. asteroides						
		N. brasiliensis						
		N. carnea						
		N. nova						
		N. otitidiscaviarum	■					
		N. paucivaurans						
		N. mexicana						
		N. tranvalensis						
		N. vinacea						
