# Supplementary material for: The Nocardia cyriacigeorgica GUH-2 genome shows ongoing adaptation of an environmental Actinobacteria to a pathogen’s lifestyle
Source: BMC Genomics. 2013 Apr 27;14:286. doi: 10.1186/1471-2164-14-286 (PMC3751702; doi:10.1186/1471-2164-14-286)
Supplement: Additional file 12 — OmniLog® metabolic profilings of N. cyriacigeorgica GUH-2 cells. Functional predictions made from genome DNA sequence analyses are indicated. Utilization of various carbon and nitrogen sources, and osmolite resistances were investigated. Differences in the data sets are indicated in grey. [file 1471-2164-14-286-S12.pdf]

Primers name	Sequence	Target gene	Tm	Fragment length	Primers name	Sequence	Target gene	Tm	Fragment length
IG1F	GAGCTGATGTCCTTCAGCTC	nocyr_0049	61	1478	IG13F	GAACTTTTCAGGACGTGCTC	nocyr_2868	61	1365
IG1R	CTATCGGCGGTGATTACTAC	nocyr_0050			IG13R	GAGACATCATCTCTACTGAC	nocyr_2869		
IG1rtF	TCGAGCGCCTGCCACGGGAGAG	nocyr_0063	63	492	IG13rtF	GCGCGGCAGCACCAATA	nocyr_2882	63	1035
IG1rtR	CGAACGGGCTGGGCATGACGAACC	nocyr_0064			IG13rtR	GGAATGCAGCGATTAAATAGTCAT	nocyr_2884		
IG2ltF	GTTATCCGGGTCTGCTGCAC	nocyr_0181	61	2660	IG14ltF	GCACACGCGAACTCGGTCTCCA	nocyr_2932	63	859
IG2ltR	GTTCGACGTGCGTTATCTGC	nocyr_0183			IG14ltR	CGTCGCGGCGTCTGAAGC	nocyr_2934		
IG2F	GAAGGCCACTAAAGTTCTCG	nocyr_0197	61	1573	IG14F	GAGCTCACTTCATTGACCAG	nocyr 2962	61	1220
IG2R	GCTGTCTTTCGCTCTGATTC	nocyr_0200			IG14R	GATCAAGATTATGGGCAGCG	nocyr_2963		
IG2rtF2	GCTGCGGTGGCCGAGGTCTATTAC	nocyr_0204	63	1423	IG14rtF	GGACGGCGGATGCAAGGAAACTGG	nocyr_2985	63	1185
IG2rtR2	GTCATCCGGATCTGGGAACACGAG	nocyr_0206			IG14rtR	AGGTGCTCGGCGGATACGGCTACA	nocyr_2986		
IG3ltF	CGACGGCCGACACCCTGACAGT	nocyr_0299	63	729	IG15ltF	CTGTCTTGGCCGCCTCGATGATGG	nocyr_3905	63	523
IG3ltR	CTGGCCCTGGTCGGTGCGGAAGTG	nocyr_0300			IG15ltR	CCTGGTCCGGCTCGCATTCTCG	nocyr_3906		
IG3F	CGATCAAGTTCCCCGACTTC	nocyr_0300	61	1583	IG15F	CATCACCTTCTCGTTGTTCG	nocyr_3909	61	1573
IG3R	CGATCTCGATCCGGATCTTG	nocyr_0301			IG15R	CGAAGCTGATCCAGGAATTG	nocyr_3910		
IG3rtF	CGGTGCGCGGGCTGAGAAGAT	nocyr_0308	63	800	IG15rtF	TCGCCGGGATGTTGTCACC	nocyr_3911	63	616
IG3rtR	TCCGGCCCCGACCTGTTGTAAGC	nocyr_0310			IG15rtR	AGCGCGGCCTGGGTCAT	nocyr_3912		
IG4ltF	GACGCGGTGCTGGGCGAATCGTT	nocyr_0339	63	673	IG16ltF2	GCGCGACGATCTTGAGGTTCTTAC	nocyr_3939	63	1415
IG4ltR	CGCGGGCTTGCGCCAGTTATCC	nocyr_0440			IG16ltR2	CGAAACTCCAGCCCGCCAGATACC	nocyr_3940		
IG4F	GAAGTTCACCTTTGCCGAAC	nocyr_0342	61	1414	IG16F	GAAGGTGTTCTATTCGCTGC	nocyr 3941	61	1235
IG4R	CGTTGGTGTTCGTGAACATG	nocyr_0344			IG16R	GCACCGAGTTCGATCAGAAC	nocyr_3943		
IG4rtF	GTGCCGAACAGGGTCGAATAGAAC	nocyr_0382	63	699	IG16rtF	ACGGGCCGGGCAGCGTCTCGT	nocyr_3962	63	859
IG4rtR	CCCACCGGGCACCTCACCAATA	nocyr_0384			IG16rtR	CGCCGCCAGCGGAAACACT	nocyr_3963		
IG5ltF	TCATCCCGCTCTTCGCCCTCCTCA	nocyr_0471	63	788	IG17ltF	ACGAGCCCAGTAGATGCCCTTGAC	nocyr_4001	63	764
IG5ltR	GCCCACCTTGCCGATCCCCTTGTC	nocyr_0473			IG17ltR	ATGGTCGCACGTCGGGAAAGTTCT	nocyr_4002		
IG5F	CAATCGATAACCGATCGAGC	nocyr_0486	61	1400	IG17F	CTTCCTTCTTCGCCATTTCG	nocyr_4013	61	1440
IG5R	GAATGTGAGGTCTTCTCCTC	nocyr_0488			IG17R	GCAACACCGCAATCGAATTC	nocyr_4015		
IG5rtF	AGGGTGGTGGCGGCGGTGTTGTAG	nocyr_0487	64	1127	IG17rtF	GCCTGGGCGGGCGGTTCTT	nocyr_4035	63	624
IG5rtR	CGAATCTGGCGCGGTGAAGGTGAG	nocyr_0489			IG17rtR	GTCCAGCCGATCCGGTTGCGTTAC	nocyr_4036		
IG6extF	GGTACTACCGATTCCGGATC	nocyr_0555	61	1232	IG18ltF	CCGATGCGGCGACCGATCTCTTGA	nocyr_4096	63	561
IG6extR	CTCCACGGCGACAAACAACG	nocyr_0557			IG18ltR	ACAGCCGCACCAGGCCGAAACAGA	nocyr_4097		
IG6F	GAGTGTTGTCGGCGTGTTTG	nocyr_0556	61	1354	IG18F	CCGAATATCGCCATTTGGTG	nocyr_4111	61	1620
IG6R	GAATATCGAGGATTTGACCG	nocyr_0557			IG18R	GTTGAACGAATCGCACATCC	nocyr_4115		
IG6rtF	CAGCGGCCCGGTACCCCGTGTT	nocyr_0560	63	1151	IG18rtF2	CCGACATCACCGGCGGCAAAGATT	nocyr_4162	63	945
IG6rtR	GACCGACATGCGTCCCAAGAGC	nocyr_0562			IG18rtR2	TTCCCGGTCCCCACGTGGTGTAAC	nocyr_tRNA32		
IG7ltF	GCGCAGTACTACCAACGGAAGAGC	nocyr_0919	63	1403	IG19ltF3	CCAGCTGCGCCGGGGACAA	nocyr_4800	63	1041
IG7ltR	GGCGCCCGAGGTGAATGTTT	nocyr_0920			IG19ltR3	CGGCCGGCGACAGCAGATAGC	nocyr_4801		
IG7F	GAATACCGGATCTACCGGATC	nocyr_0922	61	1280	IG19F	GCAAACACGGATCAACATCC	nocyr_4802	61	1373
IG7R	CAATGCCGTAATCTCGGCATC	nocyr_0923			IG19R	CTGGAATCAAGGAAGGGATC	nocyr_4804		
IG7rtF	ACCGCCGGCGCCCCTTGACA	nocyr_0924	63	450	IG19rtF	CATCGCGGTGCGGCTGCTGGACA	nocyr_4817	63	1385
IG7rtR	ACCTCGATCCCCCACACCCACTTG	nocyr_0926			IG19rtR	AGGCCCACGCCGCATTCGGTTTGA	nocyr_4819		
IG8ltF	CGTCGTCGCCTGCGTGGTGAA	nocyr_0966	63	477	IG20ltF	TGGAGCGCCCCGACAAGT	nocyr_5026	63	1175
IG8ltR	ACGGCCCGAGCTGAGGTGTCT	nocyr_0967			IG20ltR	CGGTGGGGAACAGCAGCGACAGTA	nocyr_5027		
IG8F	GCCCATCAGATCCTAATCAG	nocyr_0973	61	1448	IG20F	CATTTCGACTCATCTCGGAG	nocyr_5031	61	1427
IG8R	GGCTTTCCAAGCTCTCTTTG	nocyr_0974			IG20R	CTATGTCGCCTTCCAAAACG	nocyr_5032		
IG8rtF	CCCAGTCCCGGGGCACCGAGTAAT	nocyr_0977	63	1018	IG20rtF	ACGCCGATCGGGTTCTGCTCACAC	nocyr_5036	63	521
IG8rtR	GCGCCGTGGGCAGGCACAGC	nocyr_0979			IG20rtR	ATCGGGCCGGTCTGCTGGTCTCTG	nocyr_5037		
IG9ltF	GTTCCTGCAGCGCCTGGGCATAGC	nocyr_1340	63	1188	IG21ltF	CCACCGCCGCGATGCTGAAGATGA	nocyr_5132	63	438
IG9ltR	CCGGTACGCCTCGGAGGTCTTCTA	nocyr_1341			IG21ltR	CGCGCCGGTGGTGAGGTT	nocyr_5133		
IG9F	CCATAGATTGATCGCATGCG	nocyr_1351	61	1458	IG21F	CATCATGTATTTCGGCAGCG	nocyr_5146	61	1412
IG9R	CTGGAATATCAACGGTCAGC	nocyr_1352			IG21R	CTTGGGTATCAGCGGGAATC	nocyr_5147		
IG9rtF	CGCGGCGAACTCCGGCCAGTAGGT	nocyr_1391	63	698	IG21rtF	GCCGGTGTCGCGCATCTTGAA	nocyr_5170	63	960
IG9rtR	GGTCTGCATGTGCGCGGATACAAG	nocyr_1392			IG21rtR	GCTGACGGCGCCGGTTTGTGA	nocyr_5171		
IG10ltF	CGACGTGTAAATCGAAGTAAGG	nocyr_1738	63	1533	IG22ltF	CACCGAGGAGCGCGATTCACTG	nocyr_5382	63	1054
IG10ltR	CTCGCCGTTGCGGATAGTGAAAAT	nocyr_1741			IG22ltR	GCCGAAACGACCGCCCGCCACTA	nocyr_5383		
IG10F	GTTGAGGACAGCAAGGAAAC	nocyr_1779	61	1286	IG22F	GACGAAATCGCATCCTTGAC	nocyr_5397	63	1421
IG10R	CAAGTACTCACGTTGTCCAG	nocyr_1780			IG22R	CAAACAACCAGTGCTGGCTC	nocyr_5400		
IG10extF	CGAAGTCGTTGGTGGCGTAG	nocyr_1800	61	925	IG22rtF	CGAATTCCGCGGCCGCAGATAG	nocyr_5404	63	957
IG10extR	GAAGAGTTCGTGGAGCATTC	nocyr_18001			IG22rtR	GCTGGGCCGAACGCTCCTACAT	nocyr_5405		
IG11ltF	GCCTGCGCCGCGTGTGAATG	nocyr_1931	63	1177	ISNcy2F	ACCGCACCGACGCTGGGCTGGAT	ISNcy2	64	840
IG11ltR	CCCCAATGCCCTGCGCGGAAGTGT	nocyr_1932			ISNcy2R	CGCGGGCCGGGGTCACCACT	ISNcy2		
IG11F	GAATTCACCTTCGCAACGAG	nocyr_1934	61	1581	ISNcy3F	GCCGAGACCCGCACCGACCATTTG	ISNcy2	64	352
IG11R	GGTGATTGCAGCCTTCATAC	nocyr_1937			ISNcy3R	CGCGCCGAAGAAGTTGAT	ISNcy2		
IG11extF	GGAAAGGATTCGCGATGTCC	nocyr_1939	61	1367	ISNcy4F	GACCCCGGCTGATCTGGCGTTCGT	ISNcy4	64	426
IG11extR	GTAGGCAGCCGACAGATTCG	nocyr_1940			ISNcy4R	CGGCGGGCGTGGGCGACTC	ISNcy4		
IG12ltF	GAAGGCGTGAAGATGGCAGTCAAC	nocyr_2043	63	1561	ISNfa1F	GAGCGCCGAGCAGCACCTTGGAGA	ISNfa1	64	436
IG12ltR	AACCTCCGAGCGGTACCAGGATAC	nocyr_2044			ISNfa1R	CACCTGCACCGGCTGGGACTGAAC	ISNfa1		
IG12F	GATCACTCCTATTGTGCTCG	nocyr_2078	61	1386	ISNfa2F	TGGCGAGGTCTCCGGTAGTTGGTT	ISNfa2	64	877
IG12R	CCTGCTTGTTCAATCCATCG	nocyr_2081			ISNfa2R	ACGGCGCGGTGTTGTTTGAGTCG	ISNfa2		
IG12rtF	AGCGGTGGCTCTATGCGTCAGTCA	nocyr_2082	63	1144	ISNfa5F	CGTGCAGCGCCGCGTCGTTAC	ISNfa5	64	564
IG12rtR	CGCCCGCAGTAGTGCTCATAGAAG	nocyr_2083			ISNfa5R	CGGGCCTTGGCGCGGTTGTTG	ISNfa5		
IG13ltF	CAACTGGATCGGCAAGAACATATA	nocyr_2826	63	1746					
IG13ltR	TACCTACAACCCAACCGCTAACAC	nocyr 2827							
